# Nitrooxylation in Organic Synthesis: From Classical Nitrate Ester Formation to Modern Catalytic Strategies

**DOI:** 10.1002/chem.71302

**Published:** 2026-06-23

**Authors:** Jayanta Dey, Dmitry Katayev

**Affiliations:** ^1^ Department of Chemistry, Biochemistry, and Pharmaceutical Sciences University of Bern Bern Switzerland

**Keywords:** nitrate esters, nitrate radical, nitrooxylation, NO_x_ chemistry, *O*‐Nitration

## Abstract

Organic nitrate esters (R–ONO_2_) are structurally and electronically distinct from nitro compounds, featuring a polarized C–O–NO_2_ linkage with an intrinsically weak O–N bond. This characteristic imparts unique reactivity, enabling controlled bond cleavage and rendering nitrate esters valuable in energetic materials, pharmaceuticals (as nitric oxide (NO) donors), and as synthetic intermediates. Nitrooxylation, the installation of the –ONO_2_ group, has historically relied on harsh mixed‐acid systems (e.g., nitric acid/sulfuric acid), which suffer from poor selectivity, limited functional‐group tolerance, and significant safety concerns. Recent advances have shifted the field toward milder and more controlled approaches. Catalytic systems, bench‐stable nitrooxy transfer reagents, and alternative activation modes have improved selectivity and operational safety. In particular, photochemical, electrochemical, and mechanochemical strategies enable the in situ generation of reactive nitrogen oxide species (e.g., NO_2_•, NO_3_•) under controlled conditions, expanding the scope to include alkene difunctionalization, C(sp^3^)–H functionalization, and selective *O*‐nitration of alcohols. This review provides a comprehensive overview of the evolution of nitrooxylation chemistry, from classical methods to modern synthetic platforms. Emphasis is placed on mechanistic understanding, reagent design, and the interplay between electronic structure and reactivity, with the aim of guiding future developments toward safe, selective, and sustainable nitrate ester synthesis.

## Graphical abstract

Nitrooxylation—the installation of nitrate esters (–ONO_2_) has evolved from classical mixed‐acid protocols to catalytic and mechano‐, flow‐, photo‐, and electrochemical strategies. This review delineates ionic and radical manifolds for C–ONO_2_ bond formation, emphasizing mechanistic control, selected substrate scope, and selectivity. Applications in pharmaceuticals, energetic materials, and sustainable synthesis are highlighted.

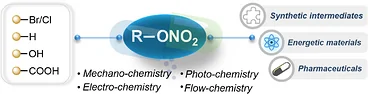

## 1 Introduction

At a structural level, organic nitrate esters (R–ONO_2_) occupy a distinctive position among nitrogen–oxygen functional groups. In contrast to the molecular orbital of the nitro compounds [1] (R–NO_2_), where the nitrogen atom is directly bonded to carbon within a strongly delocalized π‐system, nitrate esters feature a C–O–NO_2_ connectivity in which the organic fragment is linked to nitrogen via oxygen. This seemingly subtle topological difference gives rise to a fundamentally different electronic and geometric framework.

The structural parameters depicted in Figure 1a clearly reflect this distinction. The two N–O bonds within the nitro fragment (≈1.26 Å) are short and nearly equivalent, consistent with significant π‐delocalization across the NO_2_ unit. In contrast, the O–N bond connecting the nitrate group to the alkoxy oxygen is elongated (≈1.36 Å), indicating reduced bond order and a predominantly σ character. The adjacent C–O bond (≈1.43 Å) remains characteristic of a standard single bond. The angular features further emphasize this asymmetry: the O–N–O angle (∼125°) supports a trigonal planar geometry around nitrogen, whereas the C–O–N angle (∼105°) reflects a bent arrangement at the bridging oxygen, indicative of suboptimal orbital overlap along the C–O–N linkage [2]. These geometric features translate directly into an electronically polarized bonding motif. The O–N bond is embedded within a delocalized NO_2_ π‐system but lacks the stabilizing π‐interaction present in O–NO_2_ bonds. As a consequence, electron density is unevenly distributed across the nitrate framework, with the bridging O–N bond representing the weakest link. From an orbital standpoint, the lowest unoccupied molecular orbital is largely localized on this O–N unit and possesses significant antibonding character, making it particularly responsive to electron injection. In parallel, the highest occupied orbitals are dominated by oxygen lone pairs with partial antibonding contribution along the same bond axis.

**FIGURE 1:**
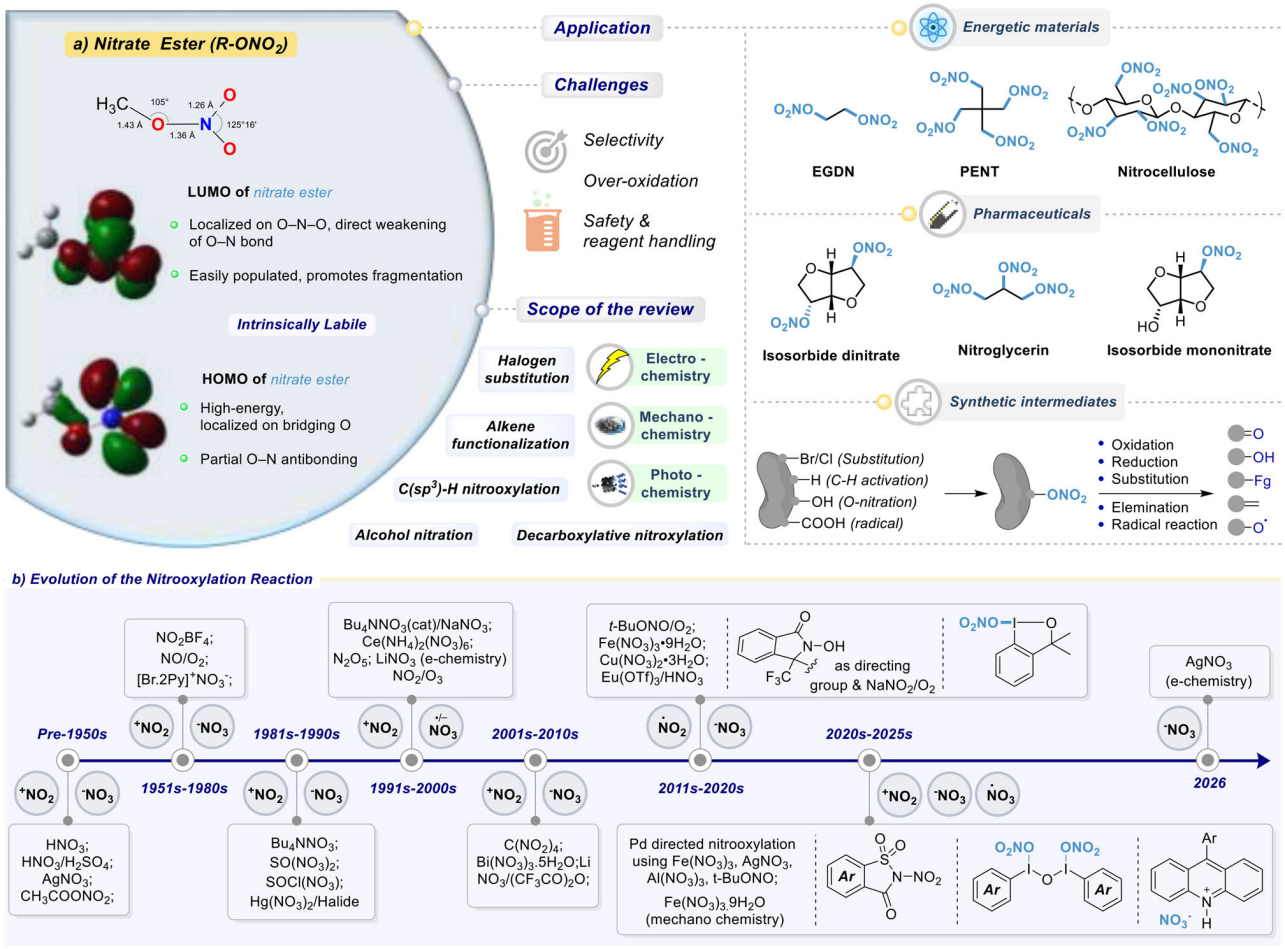
(a) Molecular orbital representation and structural features of nitrate ester & major applications; Key challenges in nitrate ester chemistry; Scope of nitrooxylation strategies including alcohol nitration, alkene nitrooxylation, and C(sp^3^)–H functionalization under classical, electrochemical, photochemical, and mechanochemical conditions. (b) Chronological progression of nitrooxylation reaction development.

The combination of bond elongation, angular distortion, and antibonding orbital character renders the O–N bond intrinsically labile. This structural bias is central to the behavior of nitrate esters across chemical contexts, governing their propensity for bond cleavage and underpinning both their synthetic utility and their inherent instability. These structural attributes directly translate into their functional roles across multiple domains. In energetic materials, polynitrate esters such as nitroglycerin, PETN, and nitrocellulose derive their high energy density from the exothermic fragmentation of O–N bonds into thermodynamically stable small molecules (e.g., N_2_, CO_2_, H_2_O) [3, 4, 5, 6, 7]. From a thermodynamic standpoint, the weak O–N bond acts as an energy reservoir, while the formation of strong N≡N and C = O bonds drives the overall process. In biological systems, the same bond polarization enables controlled nitric oxide release. Nitrate esters function as NO donors under enzymatic or thiol‐mediated conditions [8], a property that underpins the pharmacological activity of vasodilators such as nitroglycerin (GTN) and isosorbide dinitrate (ISDN) [9—12]. Importantly, this behavior contrasts sharply with nitro compounds, which are typically redox‐active but do not directly serve as NO surrogates.

From a synthetic perspective, the nitrooxy group is a versatile and strategically valuable functionality. The polarized C–O–NO_2_ motif can be transformed through elimination, substitution, or reductive pathways to access alkenes, alcohols, halides, or nitrogen‐containing products [2, 13, 14, 15]. In radical manifolds, controlled O–N bond homolysis enables downstream skeletal rearrangements or remote functionalization. As such, nitrooxylation, the installation of the –ONO_2_ group, has evolved from a niche transformation into a platform for molecular diversification (Figure 1).

The development of the nitrooxylation strategy over the years (Figure 1b) reflects a broader evolution from empirically driven, harsh transformations to increasingly controlled, mechanistically guided strategies. Early approaches were dominated by strongly acidic, oxidizing conditions, typified by mixed‐acid systems [13, 16, 17], which enabled access to nitrate esters but suffered from limited selectivity, poor functional‐group tolerance, and significant safety concerns [18]. These methods relied on highly reactive electrophilic nitrogen species, rendering them effective yet difficult to regulate, particularly in complex molecular systems.

Subsequent efforts introduced alternative nitrating agents designed to moderate reactivity and improve operational handling. Although these reagents provided incremental improvements, their intrinsic oxidative strength and sensitivity often left many of the limitations of classical protocols intact. As a result, the direct and selective installation of the nitrooxy group, especially onto multifunctional substrates, remained a persistent challenge [12, 13, 19].

A conceptual shift emerged with the exploration of complementary bond‐forming strategies, including substitution processes, ring‐opening reactions, and radical‐based additions. These approaches expanded the synthetic repertoire of nitrooxylation, albeit frequently through indirect disconnections rather than direct functionalization of native functionalities. Nevertheless, they established important precedents for engaging nitrogen–oxygen species across a broader range of reactivity manifolds [13, 14]. More recently, the field has transitioned toward catalytic, modular platforms that enable greater control over reactivity. In this context, the design of bench‐stable nitrooxy transfer reagents and the use of tailored catalytic systems have allowed nitrooxylation to proceed under comparatively mild conditions with improved selectivity [20, 21, 22, 23, 24]. Mechanistically, these advances reflect a shift from purely ionic pathways to hybrid or radical processes, in which the generation and interception of NO_x_ intermediates can be regulated in both time and energy. Concurrently, the emergence of photochemical, electrochemical, and mechanochemical methodologies has further redefined the landscape of nitrooxylation processes. These approaches decouple the generation of reactive nitrogen–oxygen species from harsh reagent conditions, enabling access to new reaction manifolds, such as alkene difunctionalization and C(sp^3^)–H functionalization, under milder, more sustainable conditions [25].

Against this backdrop, the present review summarizes the evolution of nitrooxylation from classical methods to modern catalytic and activation modes (Table 1). Emphasis is placed on the interplay between mechanism, reagent design, and selectivity, highlighting how advances in radical and redox chemistry have transformed nitrooxylation into a more predictable and versatile synthetic strategy.

**TABLE 1:** Summary of the Review.

*Section*	*Nitrooxylation strategy*	*Activation mode*
2.1	Alcohol *O*‐nitration	Classical electrophilic nitration
2.2	Nitrate substitution reactions	S_N_1/S_N_2 substitution
2.3	Ring‐opening nitrooxylation	Lewis acid or ionic activation
2.4	Alkene nitrooxylation via halonium intermediates	Electrophilic halogenation
3	Nitrooxylation of aryldiazoacetates	Carbene transfer
4.1	Cerium nitrate systems	Radical–polar crossover
4.2	Copper nitrate systems	Lewis acid‐assisted halonium pathway
4.3	Hypervalent iodine‐mediated C–H nitrooxylation	Electrophilic nitrooxy transfer
4.4	Hypervalent iodine‐mediated alkene nitrooxylation	Ionic iodonium activation
4.5	Catalytic *O*‐nitration	Lewis acid cooperative activation
5	Decarboxylative nitrooxylation	Oxidative radical decarboxylation
6	NO_2_/NO_3_ radical‐driven nitrooxylation	Radical chain process
7	Enantioselective SOMO Catalysis	Organocatalytic single‐electron activation
8	C(sp^3^)–H Nitrooxylation	HAT‐mediated/Pd‐directed functionalization
9	Photochemical nitrooxylation	Photoredox catalysis
10	Electrochemical nitrooxylation	Anodic oxidation
11	Mechanochemical nitrooxylation	Mechanical activation
12 & 13	Limitations, Safety, and Practical Considerations; Outlook and Future Directions	

## 2 Conventional Ionic Approaches to Nitrooxylation

Historically, nitrooxylation has been conceptualized within the framework of electrophilic nitration and nucleophile‐assisted substitution, or a strain‐release ring‐opening reaction with the nitrate ion, in which reactivity is governed by polar, two‐electron processes rather than radical pathways. In these systems, the nitrate fragment is not intrinsically electrophilic; instead, it is generated or delivered alongside a highly activated nitrogen‐centered electrophile (formally NO_2_^+^ equivalents), and the O–NO_2_ bond is formed via nucleophilic addition or ion‐pair recombination. Consequently, bond construction is dictated by classical parameters, carbocation stability, ion pairing, and the availability of activated electrophilic nitrogen species. Mechanistically, these transformations proceed through well‐defined ionic manifolds, including direct substitution at activated carbon centers, addition across π‐systems via cationic intermediates, or ring opening of strained substrates. From a thermodynamic perspective, the strong driving force stems from the formation of stable nitrate esters under highly oxidizing conditions; however, this is counterbalanced by poor kinetic discrimination, leading to overreaction and limited site selectivity. Such intrinsic limitations as harsh acidic media, low functional‐group tolerance, and lack of stereocontrol ultimately delineate the boundaries of classical approaches and motivate the development of modern radical and catalytic nitrooxylation strategies.

### 2.1 Nitration of Alcohol

Classical nitrate ester synthesis proceeds predominantly through three canonical disconnections: (i) direct nitrate esterification of alcohols, (ii) nucleophilic substitution of preactivated substrates, and (iii) ring opening of strained heterocycles. Among these, acid‐mediated esterification remains the most widely adopted approach. Typically, a mixed acid system composed of concentrated nitric and sulfuric acids is employed [2, 26], in which sulfuric acid serves as a dehydrating agent to generate the active nitrating species. The resulting medium promotes the formation of nitronium ion (NO_2_^+^), which, despite being formally associated with C‐nitration, also participates indirectly in nitrate ester formation through protonated intermediates and ion‐pair collapse. In certain cases, nitric acid itself has been employed directly for the nitrooxylation of alcohols [27, 28]. Additives such as urea and lanthanide are often introduced to suppress adventitious nitrous acid [29, 30], thereby minimizing competitive side reactions and decomposition pathways. From a mechanistic standpoint, alcohol activation proceeds via protonation, followed by nucleophilic attack of a nitrate equivalent or recombination with a tightly associated ion pair. The outcome is strongly influenced by substrate electronics: primary and secondary alcohols generally undergo smooth conversion (compounds **1** & **2**), whereas the tertiary alcohol (**3**) delivers a low yield (56%) and is prone to competing elimination or rearrangement, reflecting the delicate balance between substitution and carbocation‐mediated pathways. Notably, aromatic alcohols exhibit diminished reactivity under these conditions, highlighting the limited scope of classical protocols.

To circumvent the constraints imposed by strongly acidic media, alternative anhydrous nitrating systems have been developed. Acetyl nitrate, generated in situ from nitric acid and acetic anhydride [2, 28, 31], provides a milder electrophilic reagent with improved tolerance toward tertiary groups (compound **4** with 78% yield). Similarly, discrete nitronium sources such as nitronium tetrafluoroborate (NO_2_BF_4_) [32, 33], N_2_O_5_ gas [34], and *N*‐nitrocollidinium salts enable nitration under aprotic conditions, offering enhanced control over reactivity [13, 35]. However, their pronounced moisture sensitivity and operational hazards limit widespread adoption.

A complementary solution to these intrinsic limitations has emerged from reaction engineering rather than reagent redesign. In this context, Maiti and co‐workers demonstrated that continuous‐flow processing enables the safe and scalable synthesis of multi‐nitrate esters under otherwise hazardous nitrating conditions [36]. By confining highly exothermic transformations within microstructured reactors, efficient heat dissipation and precise control over residence time suppress thermal runaway and limit the accumulation of unstable intermediates. Mechanistically, the transformation remains rooted in classical electrophilic nitrooxylation; however, flow operation modulates reaction kinetics at the reactor level, effectively decoupling reactivity from safety constraints. This approach underscores that key challenges in nitrate ester synthesis are largely governed by process design rather than intrinsic chemical reactivity (Figure 2).

**FIGURE 2:**
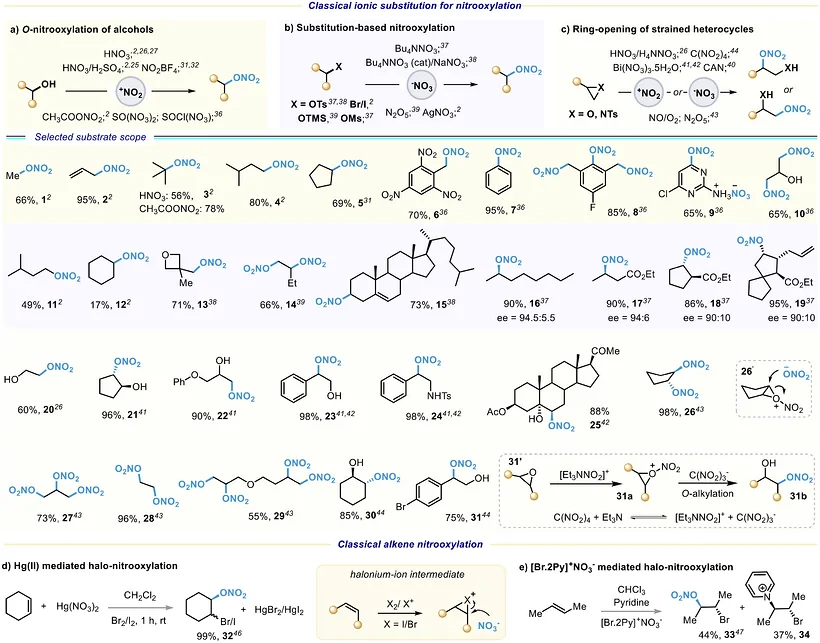
(a) O‐Nitrooxylation of alcohols via electrophilic NOx species generated from nitric or mixed‐acid systems. (b) Substitution‐based nitrooxylation of activated alkyl derivatives through S_N_1/S_N_2 pathways using nitrate salts. (c) Ring‐opening nitrooxylation of strained heterocycles (e.g., epoxides, aziridines) via nucleophilic nitrate addition. (d) Hg(II)‐mediated halo‐nitrooxylation of alkenes through electrophilic activation. (e) Halonium‐mediated alkene nitrooxylation via bromonium/halonium intermediates followed by nitrate trapping.

A notable advance was reported by Hakimelahi and coworkers, who introduced thionyl nitrate (SO(NO_3_)_2_) and thionyl chloride nitrate (SOCl(NO_3_)) as efficient nitrating agents [37]. These reagents enable rapid and selective *O*‐nitration even in the presence of aromatic π‐systems (**6**‐**9**), a context in which conventional mixed‐acid systems often fail. Aromatic alcohols such as phenols generally exhibit diminished *O*‐nitration reactivity because resonance delocalization reduces the nucleophilicity of the oxygen atom while simultaneously favoring competitive electrophilic aromatic substitution. In contrast, the high electrophilicity and mild reaction profile of thionyl nitrate‐based reagents promote rapid *O*‐functionalization, thereby suppressing competing ring nitration and delivering aryl nitrate esters in good yields. Mechanistically, these reagents likely operate through electrophilic nitrogen species coupled with in situ generation of leaving groups, facilitating clean substitution. Moreover, careful control of reagent stoichiometry enables selective mononitration of polyhydroxylated substrates such as glycerol (**10**), underscoring the role of kinetic control in suppressing overfunctionalization. Taken together, electrophilic nitrooxylation strategies highlight a recurring mechanistic theme: efficient nitrate installation is achieved at the expense of chemoselectivity and functional group compatibility. From an orbital perspective, these processes rely on strong electrophile–nucleophile interactions, with limited opportunity for fine‐tuning reactivity, whereas modern approaches allow systematic modulation of radical polarity and redox potentials.

### 2.2 Nitrate Substitution Reaction

Silver nitrate–mediated substitution of alkyl halides represents one of the earliest and operationally straightforward entries to nitrate esters within classical nitrooxylation chemistry [2]. Despite this apparent simplicity, the method is fundamentally constrained by its dependence on ionic activation pathways. Mechanistically, Ag^+^ abstracts the halide to promote C–X bond ionization, generating either a discrete carbocation or a tight ion pair. While this activation enables interception by nitrate, it concurrently opens competitive channels, most prominently solvolysis and *β*‐elimination. From a kinetic standpoint, this competition is exacerbated by the relatively low nucleophilicity of NO_3_^−^ compared to common solvent nucleophiles (e.g., H_2_O, ROH), leading to inefficient trapping of the cationic intermediate. As a result, product distributions are often mixed and yield modest results. This limitation becomes increasingly pronounced with substrate substitution: primary alkyl halides can furnish moderate yields (e.g., 47%, **11**), whereas secondary substrates exhibit a marked decline in efficiency (17%, **12**), consistent with the greater stability and thus longer lifetime of secondary carbocations, which favors side reactions over productive nitrate capture.

Efforts to circumvent these limitations have focused on enhancing the nucleophilicity of the nitrate source and improving leaving‐group ability. In this context, Cainelli and co‐workers (1983) demonstrated that enantioenriched alcohol derivatives bearing activated leaving groups (e.g., tosylates (‐OTs), mesylates (‐OMs)) undergo efficient nucleophilic substitution with nitrate salts such as tetrabutylammonium nitrate (TBAN) (1.5 equiv) at very high temperature and pressure [38]. Notably, these transformations proceed with inversion of configuration, consistent with an S_N_2 pathway, and afford nitrate esters in high yields (e.g., **16–19**). Mechanistically, the use of a phase‐transfer‐compatible nitrate source and a better leaving group shifts the reaction manifold away from carbocationic ionization toward concerted displacement, thereby suppressing rearrangement and elimination pathways.

Subsequently, Hwu, Yang, and co‐workers reported a related approach employing catalytic tetrabutylammonium nitrate and NaNO_3_ under biphasic conditions (benzene/water, 110–135°C), enabling substitution to afford nitrate ester **13** in 71% yield [39]. Although described as catalytic with respect to the nitrate source, the process likely operates via phase‐transfer‐mediated delivery of NO_3_^−^, again favoring a substitution pathway over full ionization.

In a complementary strategy, vicinal diols were converted to cyclic silyl intermediates (via dichlorodimethylsilane), which, upon treatment with excess N_2_O_5_, furnished the corresponding dinitrates (e.g., **14**) in 66% yield, although the requirement for a superstoichiometric reagent underscores the limited efficiency and practicality of this approach [40].

### 2.3 Ring‐Opening of Strained Heterocycles

In the early development of epoxide‐based nitrooxylation, Ingham and co‐workers (1953) reported one of the first ring‐opening strategies, employing ammonium nitrate in concentrated nitric acid to convert ethylene oxide into the corresponding *β*‐nitrooxy alcohol (e.g., **20**) in moderate yield (∼60%). This transformation proceeds under strongly acidic conditions, where epoxide activation occurs via protonation, followed by nucleophilic capture by nitrate. While conceptually straightforward, the method suffers from limited chemocontrol and harsh reaction media, which restrict substrate scope (Figure 2) [27].

Subsequently, Iranpoor and co‐workers introduced a more controlled variant using ceric ammonium nitrate (CAN, Ce(IV)) as a catalytic or stoichiometric promoter. In combination with external nitrate sources such as NH_4_NO_3_ or tetrabutylammonium nitrate, CAN enables efficient conversion of epoxides to *β*‐(nitrooxy)‐substituted alcohols (e.g., **26**) [41]. Mechanistically, these systems likely operate via single‐electron or Lewis acid activation of the epoxide, although the precise balance between SET and ionic pathways remains substrate‐dependent. Notably, the use of CAN mitigates some limitations of classical acid‐mediated protocols, but still relies on strongly oxidizing conditions.

A significant advance was reported in 2007 by Das and co‐workers, alongside Salvador and collaborators, who demonstrated that bismuth(III) nitrate enables the efficient ring‐opening of oxiranes and aziridines under mild conditions to furnish *β*‐(nitrooxy)‐substituted alcohols and amines (**21–25**). These transformations proceed with high regio‐ and stereoselectivity and avoid the use of strongly acidic media [42, 43]. However, a distinct inversion of regioselectivity is observed for styrenyl substrates. In the case of styrene oxide or styrene aziridine, nucleophilic attack preferentially occurs at the benzylic position, yielding products **23** and **24**. Mechanistically, this behavior reflects Lewis‐acid coordination of Bi(III) to the epoxide (or aziridine), thereby enhancing C–O (or C–N) bond polarization. The resulting transition state acquires significant carbocation character, and the benzylic carbon is selectively activated through π‐stabilization. Given the intrinsically weak nucleophilicity of nitrate, capture occurs at the more electrophilic site, placing these reactions along an S_N_2–S_N_1 continuum rather than a purely concerted displacement pathway. Thus, 2‐aryl epoxides function as sensitive probes of electronic control in nitrooxylation chemistry.

In parallel, Millar and coworkers (1993) developed a complementary strategy for di‐ and polynitrooxylation via epoxide ring opening using dinitrogen pentoxide (N_2_O_5_) [44]. Under these conditions, N_2_O_5_ undergoes heterolytic dissociation to generate NO_2_^+^ and NO_3_^−^, establishing a highly electrophilic environment. Mechanistically, NO_2_^+^ activates the epoxide through electrophilic interaction, facilitating ring opening and subsequent trapping by nitrate to afford dinitrated products (as shown **26′**). In substrates such as glycidol, sequential activation and capture enable formation of trinitrooxylated products (**27**), whereas polyepoxides undergo multiple ring‐opening events to furnish higher‐order nitrooxylated compounds (e.g., **29**). Although highly effective for dense functionalization, these processes are intrinsically driven by strong ionic activation and therefore lack the selectivity and functional group tolerance characteristic of more modern catalytic systems. A related yet conceptually distinct approach was later reported by Averina and coworkers, who employed tetranitromethane C(NO_2_)_4_ in the presence of triethylamine to achieve epoxide ring opening to *β*‐hydroxy nitrates [45]. The method enables efficient formation of nitrate esters (e.g., **30**, **31**) with good regioselectivity and functional‐group tolerance relative to classical acid‐mediated systems. Mechanistically, the transformation proceeds via base‐assisted activation of C(NO_2_)_4_, generating a reactive ion pair ([Et_3_NNO_2_]^+^ / C(NO_2_)_3_^−^) (see **31′**). Nucleophilic attack of the trinitromethyl anion on an epoxide‐derived oxonium intermediate (**31a**) furnishes a transient nitronate species (**31b**), which subsequently undergoes solvolytic fragmentation to deliver the *β*‐hydroxy nitrate.

Collectively, these studies delineate the evolution of epoxide‐based nitrooxylation from strongly acidic, ionically driven processes to more controlled Lewis acid‐mediated strategies, with regioselectivity increasingly governed by substrate electronics and transition‐state polarization rather than simple steric effects.

### 2.4 Alkene Nitrooxylation via Halonium Intermediates

In early studies of alkene nitrooxylation, radical and ionic pathways often coexisted, leading to limited selectivity and complex product profiles. In this context, Brown (1956) reported one of the earliest examples of nitrooxylation of isobutylene using nitric oxide. The reaction, however, proceeded via a poorly controlled radical manifold, yielding a mixture of products arising from competing oxidation, addition, and fragmentation pathways. This lack of selectivity reflects the intrinsic challenge of controlling NO‐derived reactive intermediates, where multiple radical recombination and disproportionation processes compete under aerobic or oxidative conditions [46].

A more controlled ionic approach emerged from the work of Barluenga and coworkers, who developed a mercury(II) salt–halogen system for the stereoselective halo‐nitrooxylation of alkenes [47]. In this method, a combination of Hg(II) salts (including Hg(NO_3_)_2_) and halogens (Br_2_ or I_2_) enables the formation of 1,2‐bifunctionalized products via a highly ordered ionic pathway. Mechanistically, the reaction proceeds through initial formation of a halonium ion intermediate, followed by anti‐addition of the nitrate anion. This leads to trans‐selective incorporation of halogen and nitrate across the double bond, consistent with backside opening of the cyclic halonium intermediate. Notably, substrate‐dependent regioselectivity is observed: for styrene, the reaction delivers the Markovnikov product exclusively, whereas terminal alkenes such as hex‐1‐ene afford mixtures of Markovnikov and anti‐Markovnikov adducts, reflecting a balance between electronic stabilization and steric accessibility (as discussed in the epoxide ring opening section). This work represents a key transition toward more predictable alkene nitrooxylation, where stereochemical control is governed by well‐defined ionic intermediates. Complementary to this, subsequent studies employing bromonium–nitrate systems further reinforced the role of cyclic halonium intermediates in controlling regio‐ and stereoselectivity. In these transformations, electrophilic bromine generates a bromonium ion across the alkene, which is then intercepted by nitrate or related nucleophiles. In certain cases, pyridine or substituted pyridinium salts participate, leading to halo–pyridinium or halo‐nitrate products (e.g., **33** & **34**) [48]. Mechanistically, these reactions follow a similar trajectory: electrophilic π‐activation, formation of a bridged halonium intermediate, and regioselective nucleophilic opening dictated by carbocation stabilization (Markovnikov bias) and anti‐addition geometry.

Taken together, these early studies highlight two distinct but converging paradigms in alkene nitrooxylation: (i) uncontrolled radical addition using NO‐derived species, which suffers from poor selectivity, and (ii) ionic halonium‐mediated difunctionalization, which offers improved stereocontrol but remains dependent on strong electrophiles and often toxic metal salts. Mechanistically, the latter establishes the foundation for modern difunctionalization strategies, in which regioselectivity arises from the polarization of π‐bonds and the stabilization of the developing positive charge, while stereoselectivity is enforced by cyclic, three‐membered intermediates.

## 3 Nitrooxylation of Aryldiazoacetates

In 2019, Jurberg and coworkers reported an efficient iron(III) nitrate–mediated nitrooxylation of aryldiazoacetates [49], enabling rapid access to nitrate esters across a broad substrate scope (more than 30 examples and yields up to 99%). A range of ester substituents (**35–39**) and aryl diazo substrates bearing both electron‐donating and electron‐withdrawing groups (**40–43**) were well tolerated, typically affording the desired products in excellent yields. Importantly, comparable efficiency was observed when stoichiometric HNO_3_ (1.2 equiv) was used in place of Fe(NO_3_)_3_ (e.g., **35** and **40**), indicating that the iron salt primarily serves as a nitrate source and Lewis acid activator, rather than a redox‐active promoter.

Mechanistically, the reaction proceeds through a carbene‐based ionic pathway. Under the reaction conditions, Fe(NO_3_)_3_ undergoes partial hydrolysis to generate HNO_3_, which can protonate the aryldiazoacetate to form a highly reactive diazonium‐type intermediate (**A**). Subsequent extrusion of N_2_ furnishes an electrophilic carbene species. Alternatively, coordination of Fe^3^^+^ to the diazo substrate (intermediates **B** or **C**) can facilitate carbene formation through Lewis's acid activation, with subsequent protonation converging to the same reactive intermediate **A** (Figure 3). In the key bond‐forming step, the highly electrophilic carbene center is intercepted by nitrate, despite its weak nucleophilicity, yielding the nitrate ester. From a mechanistic standpoint, this transformation is best described as a polar carbene capture process, rather than a radical pathway.

**FIGURE 3:**
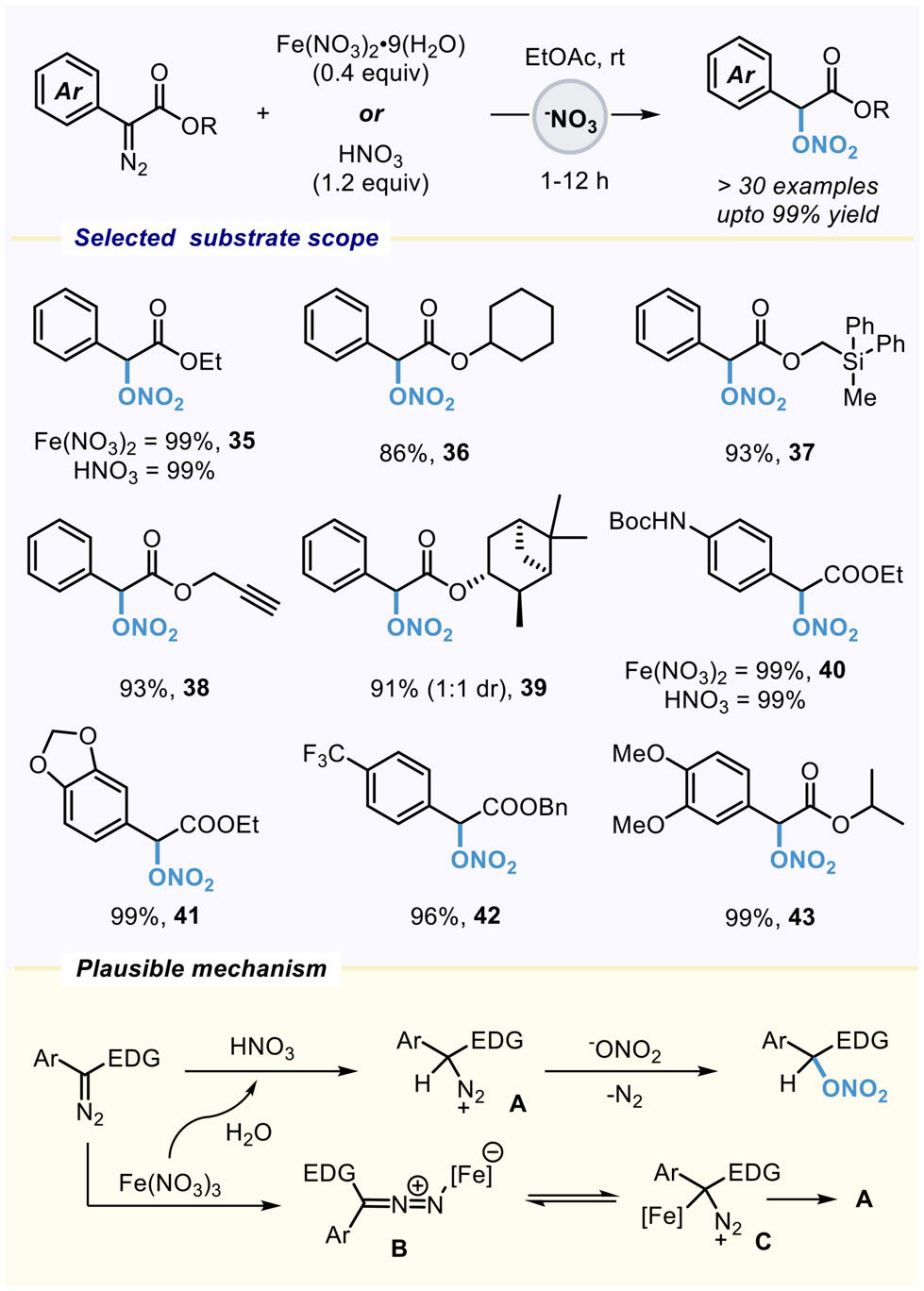
Nitrooxylation of aryldiazoacetates with Fe(NO_3_)_3_ and HNO_3_.

Consequently, Lv, Wei, Yi, and coworkers reported a metal‐free, visible‐light‐mediated nitrooxylation of α‐diazoesters, using cyclic ethers and *t*‐butyl nitrite (*t*‐BuONO) under aerobic conditions [50]. The protocol accommodates a broad range of aryldiazoacetates bearing both electron‐donating and electron‐withdrawing substituents, as well as diverse ester groups, delivering the corresponding nitrate esters in moderate to good yields. In addition, various cyclic ethers, including tetrahydrofuran (**44–50**), tetrahydropyran (**51**), and 1,4‐dioxane (**52**), were successfully incorporated, demonstrating the generality of this multicomponent transformation across more than 25 examples, with yields up to 89% (Figure 4).

**FIGURE 4:**
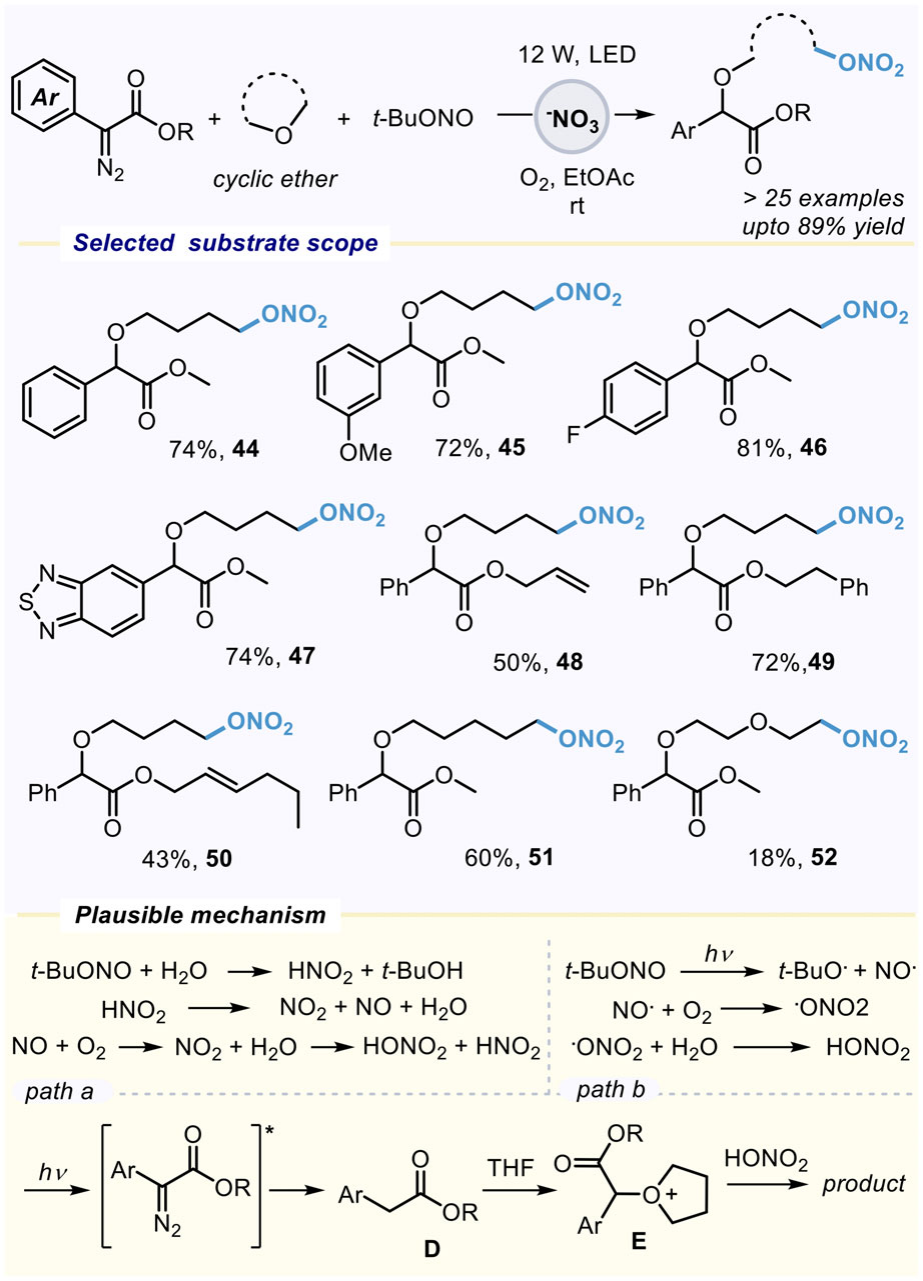
Nitrooxylation of aryldiazoacetates with *t*‐BuONO.

Mechanistic insight was obtained through a series of control experiments. The reaction is completely suppressed in the absence of light, confirming the requirement for photochemical activation, and is also significantly inhibited under N_2_ atmosphere or anhydrous conditions, establishing the essential roles of both O_2_ and H_2_O. Deuterium‐labeling experiments using D_2_O revealed incorporation at the α‐position of the ester, indicating that the proton source originates from water. Furthermore, ^18^O‐labeling studies (H_2_O^18^) showed that the oxygen atoms in the nitrate ester derive from a combination of water and molecular oxygen, consistent with in situ generation of reactive NO_x_ species. The formation of nitrate ions in the reaction medium was additionally confirmed by the brown ring test, while independent experiments demonstrated that HNO_3_ or NaNO_3_ can substitute *t*‐BuONO to furnish the same product, supporting nitrate as the active nucleophilic species. Based on these observations, the authors propose a carbene–ylide mechanism. Visible‐light excitation of the diazo compound generates an excited state that undergoes N_2_ extrusion to form a singlet carbene, which is subsequently trapped by the cyclic ether to produce an oxonium ylide intermediate. Concurrently, *t*‐BuONO participates in a NO_x_ cycle, wherein hydrolysis and oxidation processes generate HNO_3_ via NO/NO_2_ intermediates in the presence of O_2_ and H_2_O. In the key bond‐forming step, the oxonium ylide is intercepted by HNO_3_ (or nitrate), delivering the nitrooxylated product.

## 4 Reagent‐Controlled Nitrooxylation: Ionic–Radical Reactivity

Metal nitrate systems and modern reagent‐controlled platforms collectively define a versatile domain of nitrooxylation that spans both ionic and radical reactivity. While metal nitrates (e.g., Ce, Cu) typically engage in single‐electron transfer (SET) processes to generate reactive NO_x_ species, organic reagents, such as hypervalent iodine derivatives, enable highly controlled, polarity‐driven nitrate transfer via electrophilic activation. Mechanistically, these approaches form a continuum ranging from radical–polar crossover pathways, where carbon‐centered radicals are oxidized to carbocations prior to nitrate capture, to fully ionic manifolds involving halonium or iodonium intermediates. This dual reactivity framework not only improves regioselectivity and chemoselectivity but also enables nitrooxylation under milder, more tunable conditions. Together, these strategies highlight how precise control over nitrate activation mode governs the efficiency and selectivity of alkene, C‐H, and C–OH nitrooxylation reactions.

### 4.1 Cerium Nitrate Systems

A transition toward oxidatively initiated radical–polar crossover manifolds was established in cerium‐mediated nitrooxylation. In 2001, Nair and co‐workers reported a CAN‐promoted bromo‐nitrooxylation of styrenes (**53**), in which cerium(IV) ammonium nitrate (CAN) serves as both a single‐electron oxidant and a nitrate source. Although detailed mechanistic studies in this early report were limited, the observed Markovnikov‐selective nitrate incorporation and requirement for Ce(IV) strongly suggest the involvement of a cationic intermediate formed after initial oxidation [51]. A more thoroughly investigated system was later reported by Yang, Wang, and co‐workers (2018), who developed a Ce(IV)‐promoted phosphinoylation–nitratation of alkenes, delivering β‐nitrooxy phosphonates (e.g., **54–61**) in moderate to good yields (Figure 5) [52]. Mechanistically, this transformation is supported by a series of diagnostic control experiments. First, the addition of radical scavengers such as TEMPO or BHT completely suppresses product formation, indicating that radical intermediates are essential to the reaction pathway. Second, the use of a radical trap (1,1‐diphenylethylene) diverts the reaction toward a phosphine oxide–derived adduct, providing direct evidence for the intermediacy of a P‐centered radical. Together, these experiments unambiguously establish a radical initiation step via single‐electron transfer (SET) from Ce(IV). Based on these observations, the authors propose a mechanism in which CAN (E° ≈ +1.6 V vs SCE) oxidizes diphenylphosphine oxide to generate a phosphinoyl radical, which subsequently adds to the alkene to form a benzylic carbon‐centered radical. This intermediate then undergoes a second oxidation by Ce(IV) to generate a benzylic carbocation, which is finally trapped by nitrate to furnish the nitrooxy product. This sequence constitutes a prototypical radical–polar crossover pathway, in which radical addition and ionic capture are temporally separated but mechanistically coupled. Notably, the regioselectivity is consistent with this mechanistic pathway. The preferential formation of benzylic nitrate products reflects both the lower oxidation potential of benzylic radicals and the thermodynamic stabilization of the ensuing carbocation, which facilitates efficient trapping by the weakly nucleophilic nitrate anion. In contrast, when simple aliphatic alkenes are employed, competing hydrophosphination pathways dominate, further supporting the requirement for stabilized radical/cation intermediates.

**FIGURE 5:**
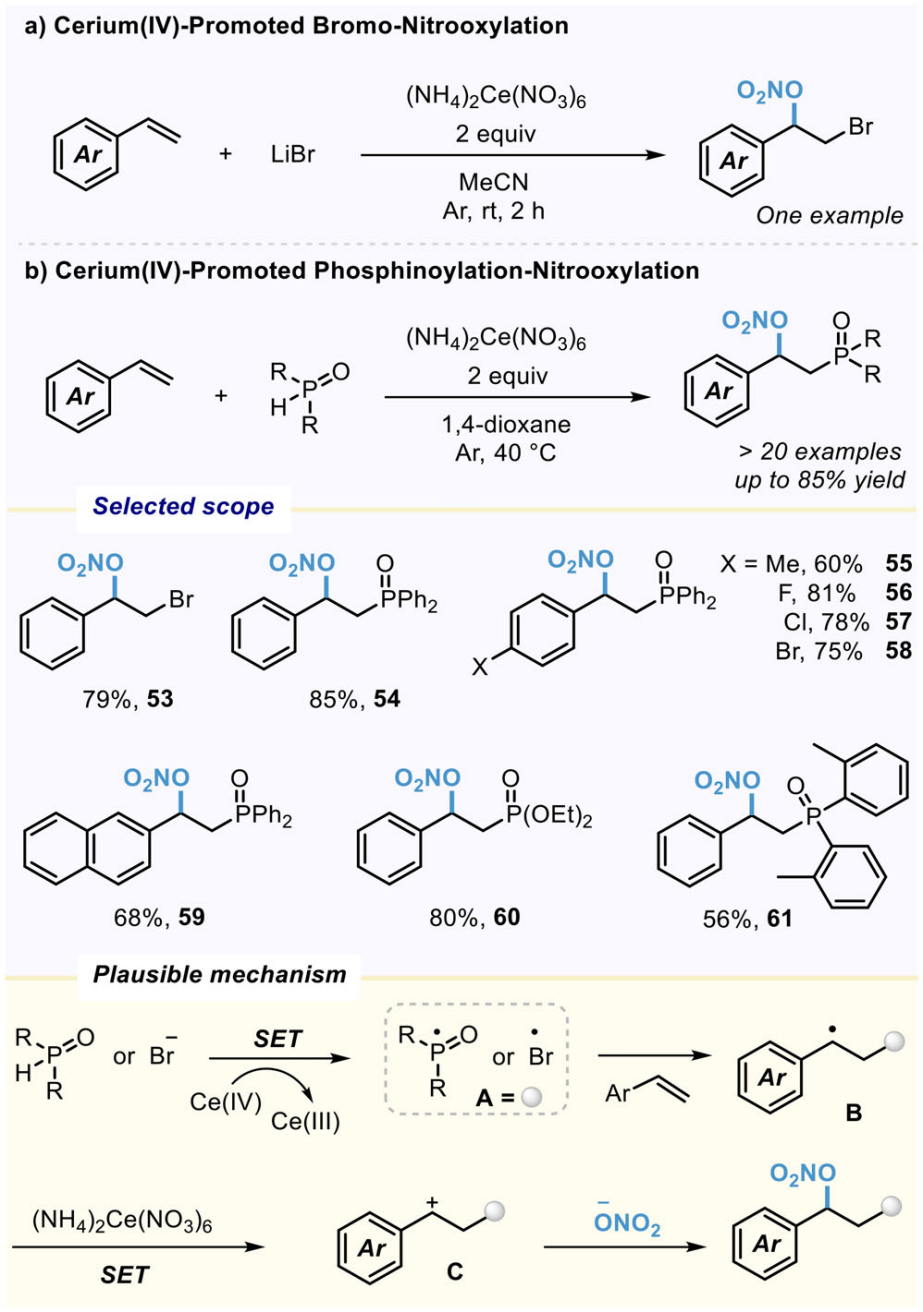
Cerium (IV) ammonium nitrate promoted nitrooxylation.

### 4.2 Copper Nitrate Systems

In 2019, Xu and co‐workers reported a copper nitrate‐mediated bromonitrooxylation of alkenes, enabling efficient access to *β*‐bromonitrates from both activated styrenyl substrates (e.g., **62–66**) and unactivated alkenes (e.g., **67**) with more than 25 examples and yields up to 92% (Figure 6) [53]. While styrenes afforded the desired products in generally high yields with good regioselectivity, unactivated alkenes displayed diminished efficiency and, in some cases, regioisomeric mixtures, reflecting the reduced electronic bias in these substrates.

**FIGURE 6:**
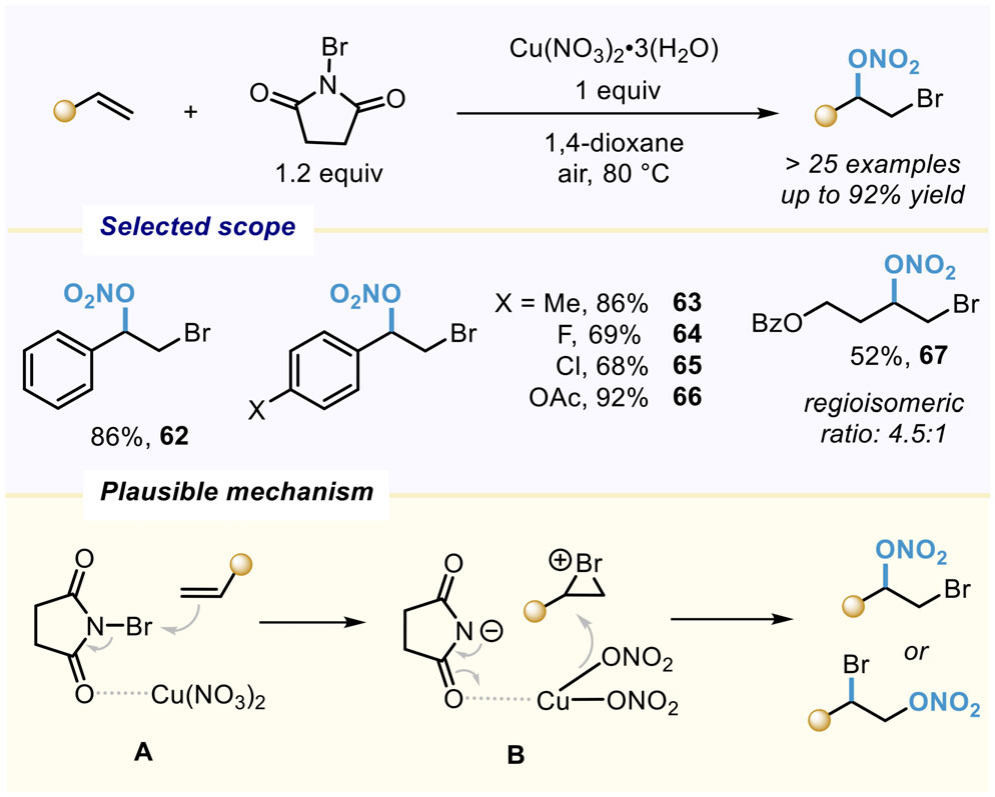
Copper (II) nitrate promoted nitrooxylation.

Mechanistically, this system differs fundamentally from the Ce(IV)‐mediated radical–polar crossover pathway and instead operates predominantly through an ionic halonium manifold. Control experiments described in the study provide important mechanistic insight. For instance, the reaction is strongly inhibited by hydroquinone, although this effect is attributed to the inhibitor's consumption by Cu(NO_3_)_2_/NBS rather than to clean radical trapping, complicating interpretation. More, revealing the radical clock experiment, which yields a ring‐opened product, but this outcome alone does not unambiguously distinguish between radical and ionic pathways, as acknowledged by the authors. Importantly, substrates prone to intramolecular radical cyclization fail to undergo such transformations, arguing against freely diffusing carbon‐centered radicals and instead supporting a concerted or tightly ion‐paired pathway.

On this basis, the authors propose a mechanism in which copper nitrate acts as a Lewis acid to activate *N*‐bromosuccinimide (NBS), generating a more electrophilic brominating species (intermediate **A**). Subsequent addition of this activated bromine source to the alkene forms a cyclic bromonium ion intermediate **B**, which is then regioselectively opened by nitrate to furnish the *β*‐bromonitrate products. The observed regioselectivity, particularly for styrenyl substrates, arises from preferential nucleophilic attack at the more substituted (benzylic) position, consistent with stabilization of the positive charge in the bromonium intermediate. From the same group, this reactivity paradigm was further expanded to a 1,3‐difunctionalization of donor–acceptor cyclopropanes using copper nitrate in combination with N‐halosuccinimides, enabling direct access to γ‐halonitrates under mild conditions. In this system, copper nitrate plays a dual role as both a Lewis acid to activate the strained cyclopropane and as a nitrooxy source, facilitating regioselective ring‐opening followed by halogenation [54].

### 4.3 Hypervalent Iodine‐Mediated C–H Nitrooxylation

The hypervalent iodine‐based nitrooxy transfer reagent employed in this transformation traces its origin to the seminal work of Kita and co‐workers (1996), who first reported the synthesis of cyclic iodine(III) scaffolds bearing nitrato ligands [55]. Katayev and co‐workers (2020) presented the use of a hypervalent iodine reagent (**1A**) for the direct nitrooxylation of enolizable C–H bonds under both Brønsted and Lewis acid activation. Two operationally distinct conditions were identified: condition A (MeCN/H_2_O), which is optimal for aryl‐substituted *β*‐keto esters, and condition B (CuOAc/MeCN/H_2_O), which is better suited for cyclic and acyclic 1,3‐dicarbonyl compounds. These complementary conditions underscore the **1A** reagent's dual activation modes, enabling efficient engagement of substrates with diverse enolization profiles [56]. From the substrate scope, the method exhibits broad applicability across *β*‐keto esters, 1,3‐diketones, and malonates. Cyclic β‐keto esters (e.g., **68–73**) undergo smooth nitrooxylation in good to excellent yields, with high chemoselectivity for the activated methylene position. Acyclic 1,3‐dicarbonyl systems are also competent substrates, although increased steric demand or reduced enolate stabilization leads to somewhat diminished efficiency. Overall, the selectivity trends are consistent with preferential formation and trapping of the thermodynamically favored enol/enolate.

Mechanistically (Figure 7), the reaction proceeds via initial enolization of the 1,3‐dicarbonyl substrate **1B**, generating a nucleophilic enol/enolate that interacts with the hypervalent iodine reagent. Activation of reagent **1A** through Brønsted or Lewis acid coordination at the oxygen atom increases polarization of the I–ONO_2_ bond, and the nucleophilic enol/enolate reacts with the activated iodine (III) species to form a transient adduct, followed by reductive elimination from iodine to forge the C–ONO_2_ bond and release the nitrate ester product **1C**.

**FIGURE 7:**
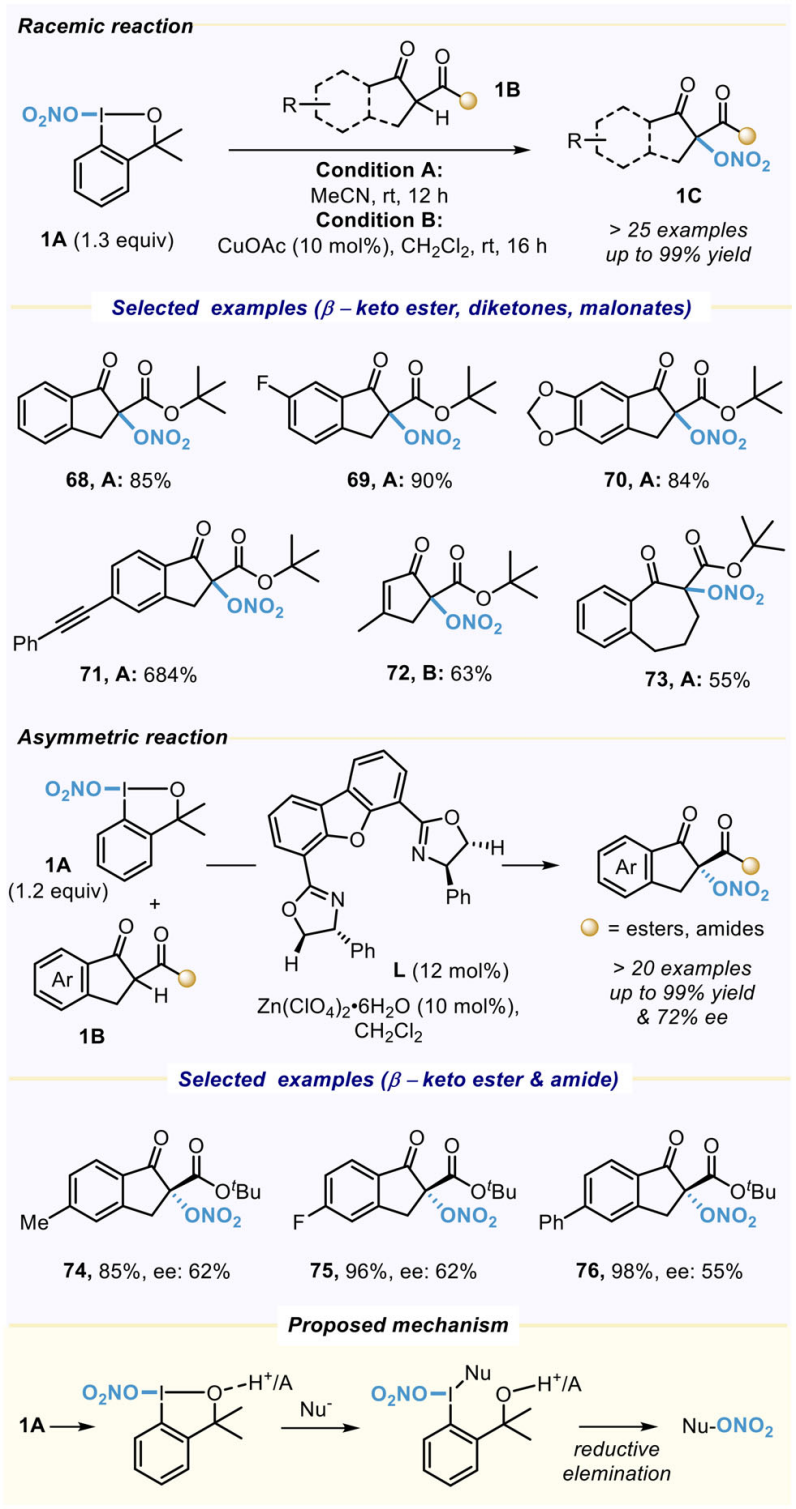
Hypervalent Iodine‐Mediated Racemic and Asymmetric C–H Nitrooxylation.

The asymmetric variant illustrated in Figure 7 represents a significant conceptual extension of the racemic manifold developed by Deng and coworkers, in which enolate generation and electrophilic nitrooxy transfer are rendered enantioselective through chiral Lewis acid control [57]. Specifically, the combination of Zn(ClO_4_)_2_ with a chiral bisoxazoline (dbfox‐type) ligand establishes a well‐defined coordination environment that binds the β‐keto ester/amidate in a bidentate fashion, enforcing a preferential enolate geometry. Within this chiral pocket, the approach of the hypervalent iodine reagent (**1A**) becomes diastereotopically differentiated, leading to facially selective C–ONO_2_ bond formation. From a mechanistic standpoint, the key stereodetermining event is the nucleophilic attack of the zinc‐bound enolate on the activated iodine(III) center. The rigid Zn–ligand scaffold biases the trajectory of this attack, effectively lowering the activation barrier for one enantiotopic face over the other. However, as reflected in the reported data (up to ∼72% ee), enantioinduction remains moderate, which can be attributed to competing background pathways and the relatively labile, weakly coordinating nature of the nitrate transfer event.

Related asymmetric variants employing chiral *N*,*N*′‐dioxide–metal complexes have been shown by Cao, Feng and coworkers to deliver *α*‐nitrooxy *β*‐keto amides/esters in up to 85% yield with enantioselectivities reaching 92% ee, highlighting the improved stereocontrol achievable with rigid chiral Lewis acid frameworks [58].

Despite the generally high yields, enantioselectivity is strongly dependent on both the substrate structure (e.g., the steric bulk of the ester group) and the chiral ligand, suggesting that the catalyst–substrate organization is not fully controlled. Mechanistically, the rapid and low‐barrier reductive elimination from the iodine(III) center limits effective stereochemical discrimination. As a result, nonselective pathways can compete, leading to only moderate enantioinduction even in well‐designed chiral systems.

### 4.4 Noncyclic Hypervalent Iodine Reagent

More recently, Deng and coworkers introduced a hypervalent iodine nitrooxylating reagent (1d) that enables rapid, mild nitrooxylation of strained substrates. Under optimized conditions (Zn(OTf)_2_, 5 mol%, CH_2_Cl_2_, rt, 1–5 min), cyclopropyl silyl ethers undergo efficient ring‐opening to furnish β‐nitrooxy ketones in moderate to good yields with broad functional group tolerance (more than 50 examples, up to 84% yield) [59].

From the substrate scope (Figure 8), aryl‐, alkyl‐, and heteroaryl‐substituted cyclopropyl silyl ethers are all competent substrates, delivering products (**77–82**) typically in ∼50–75% yield, with tolerance toward esters, halides, and complex cyclic frameworks. Mechanistically, the reaction is initiated by Lewis acid activation of the silyl ether by Zn(II), generating a coordinated intermediate that engages with the iodine(III) reagent. Subsequent formation of a cyclopropoxy–iodonium/zinc complex triggers C–C bond cleavage, producing radical intermediates that recombine with a nitrooxy fragment to deliver the product. Importantly, radical‐inhibition experiments (TEMPO, BHT) significantly suppress the reaction, and trapping experiments provide adducts, supporting the involvement of radical species rather than a purely ionic pathway, as shown in Figure 8.

**FIGURE 8:**
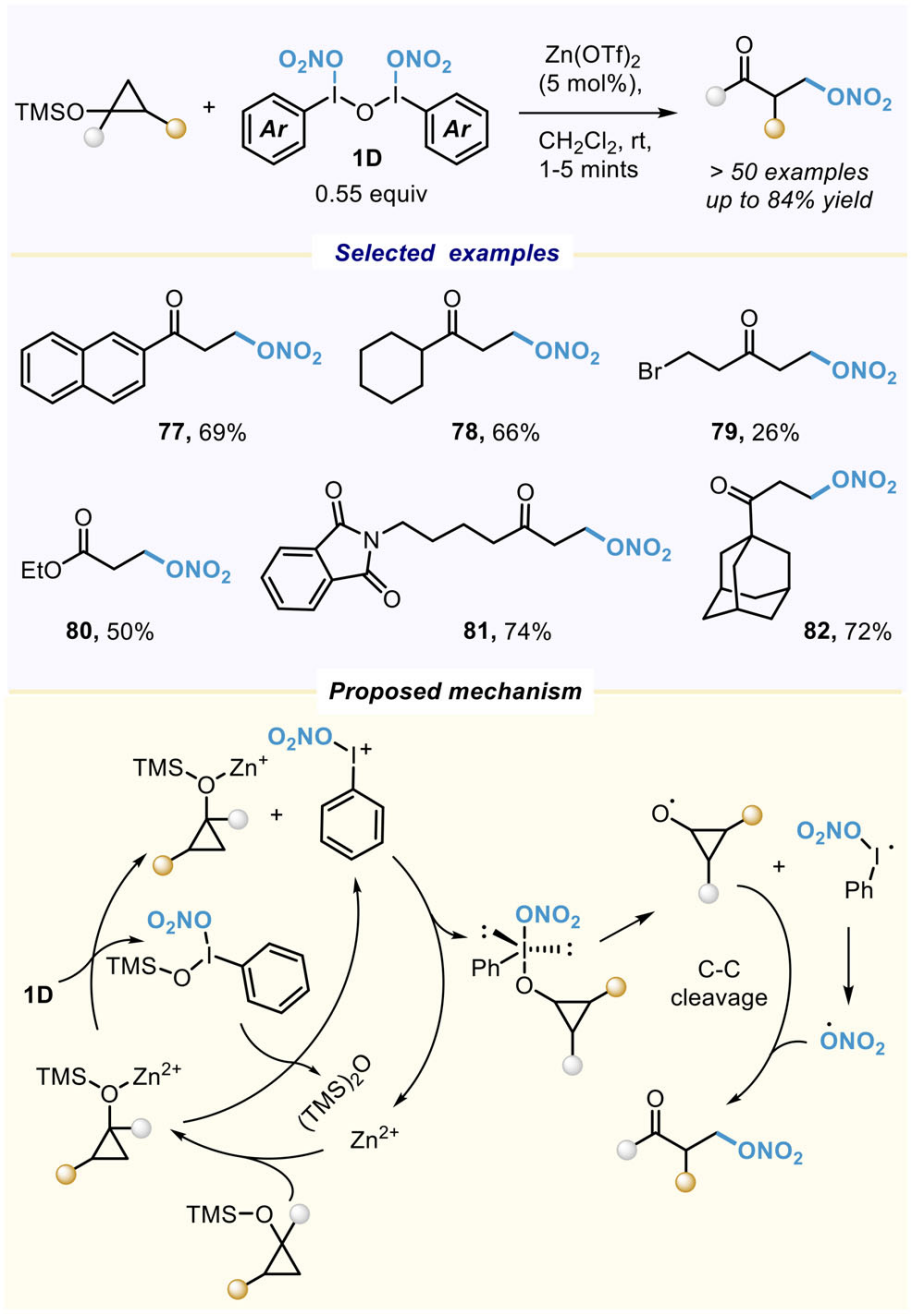
Hypervalent Iodine‐Mediated ring‐opening nitrooxylation of cyclopropyl silyl ethers.

In 2024, the same group developed a complementary ionic strategy for alkene nitrooxylation using hypervalent iodine reagents, which enable controlled oxy‐functionalization under mild conditions while avoiding transition metals. As illustrated in the substrate scope (Figure 9), the reaction employs TMSX (X = Cl, Br, I) in combination with FeCl_3_ (Condition A) or under metal‐free conditions (Condition B) to promote efficient halo‐nitrooxylation of alkenes [60].

**FIGURE 9:**
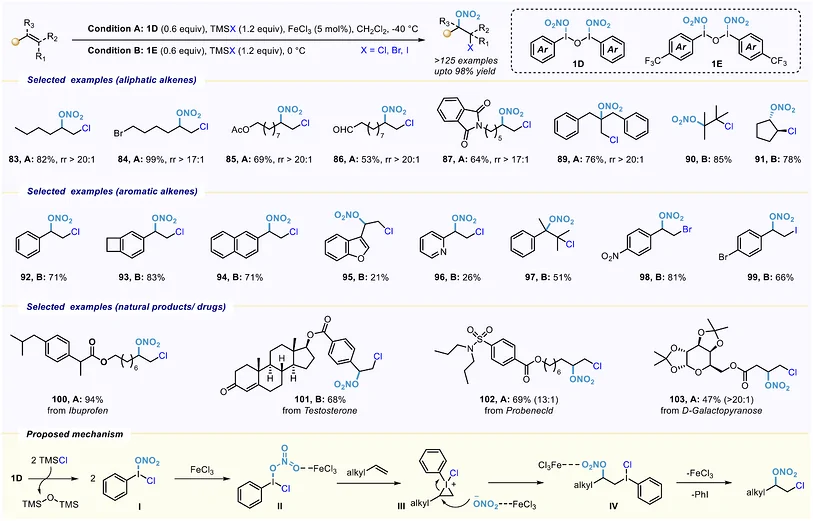
Hypervalent iodine reagents for alkene halo‐nitrooxylation.

From a scope perspective, the methodology demonstrates broad applicability to both aliphatic alkenes and styrene derivatives. For nonaromatic (aliphatic) substrates, a variety of terminal and internal alkenes undergo smooth difunctionalization to furnish β‐halo nitrate esters (**83–91**) in moderate to excellent yields (typically ∼70–85%), with good regioselectivity favoring Markovnikov‐type nitrate incorporation. Notably, functional groups such as esters, aldehydes, and cyclic frameworks are well tolerated, indicating a relatively mild and chemoselective process. In contrast, styrenyl systems exhibit enhanced reactivity and selectivity under slightly modified conditions (Condition B). Electron‐neutral and electron‐rich styrenes deliver the desired products (**92–99**) in generally higher yields, often exceeding 80%, with excellent regioselectivity for benzylic nitrooxylation.

Importantly, the methodology extends to late‐stage functionalization, as demonstrated by natural product derivatives (**100–103**), underscoring its synthetic utility. Mechanistic studies provide insight into the operative pathway. The reaction proceeds via activation of the hypervalent iodine reagent by FeCl_3_ or halide (TMSX) to generate an electrophilic iodine(III) species. Subsequent alkene coordination and formation of an iodonium intermediate (II/III) establish a polarized three‐membered ring analogous to classical halonium ions. Evidence supporting this ionic manifold includes: (i) the absence of radical inhibition effects, (ii) high stereospecificity consistent with anti‐addition, and (iii) strong regioselectivity dictated by carbocation‐like stabilization at the more substituted carbon.

In the proposed mechanism (Figure 9), the alkene reacts with the activated iodine(III) species to form a cyclic iodonium intermediate, which is then regioselectively opened by nitrate to generate intermediate IV. Subsequent halide substitution (Cl^−^/Br^−^/I^−^) or ligand exchange furnishes the final *β*‐halo nitrate product, with regeneration of the iodine catalyst. From an orbital perspective, the reaction is governed by σ‐bond activation of the C–I bond and by nucleophilic attack at the more‐substituted carbon, reflecting a balance between electronic stabilization and steric accessibility.

### 4.5 Catalytic O‐Nitration

As a further advance, Katayev and coworkers reported in 2024 a bench‐stable and recyclable *N*,6‐dinitrosaccharin reagent that, in combination with Mg(OTf)_2_ and 2‐fluoropyridine, enables mild and highly selective alcohol nitration at room temperature [61]. Conceptually, this work addresses a longstanding limitation of classical *O*‐nitration: the need for strongly acidic, often poorly chemoselective nitronium‐generating systems. In contrast, the present platform operates under catalytic Lewis's acid activation and shows unusually broad compatibility with functional groups (more than 40 examples, up to 99% yield). The substrate scope highlighted in Figure 10 shows that primary alcohols derived from aryl/alkyl scaffolds, cyclic systems, and aliphatic chains are cleanly converted to the corresponding nitrate esters, while sensitive unsaturation remains intact. The method also extends effectively to secondary alcohols, including biologically important molecules, again without substantial competing oxidation or elimination (e.g., 104–112). Particularly significant is the successful and selective nitration of diols, furnishing mono‐nitrated β‐hydroxy nitrates (114) and related hydroxy‐nitrate motifs without detectable overnitration (115). Even the synthesis of a constrained tertiary nitrate was demonstrated in the case of an adamantyl derivative (113), where elimination is structurally disfavoured.

**FIGURE 10:**
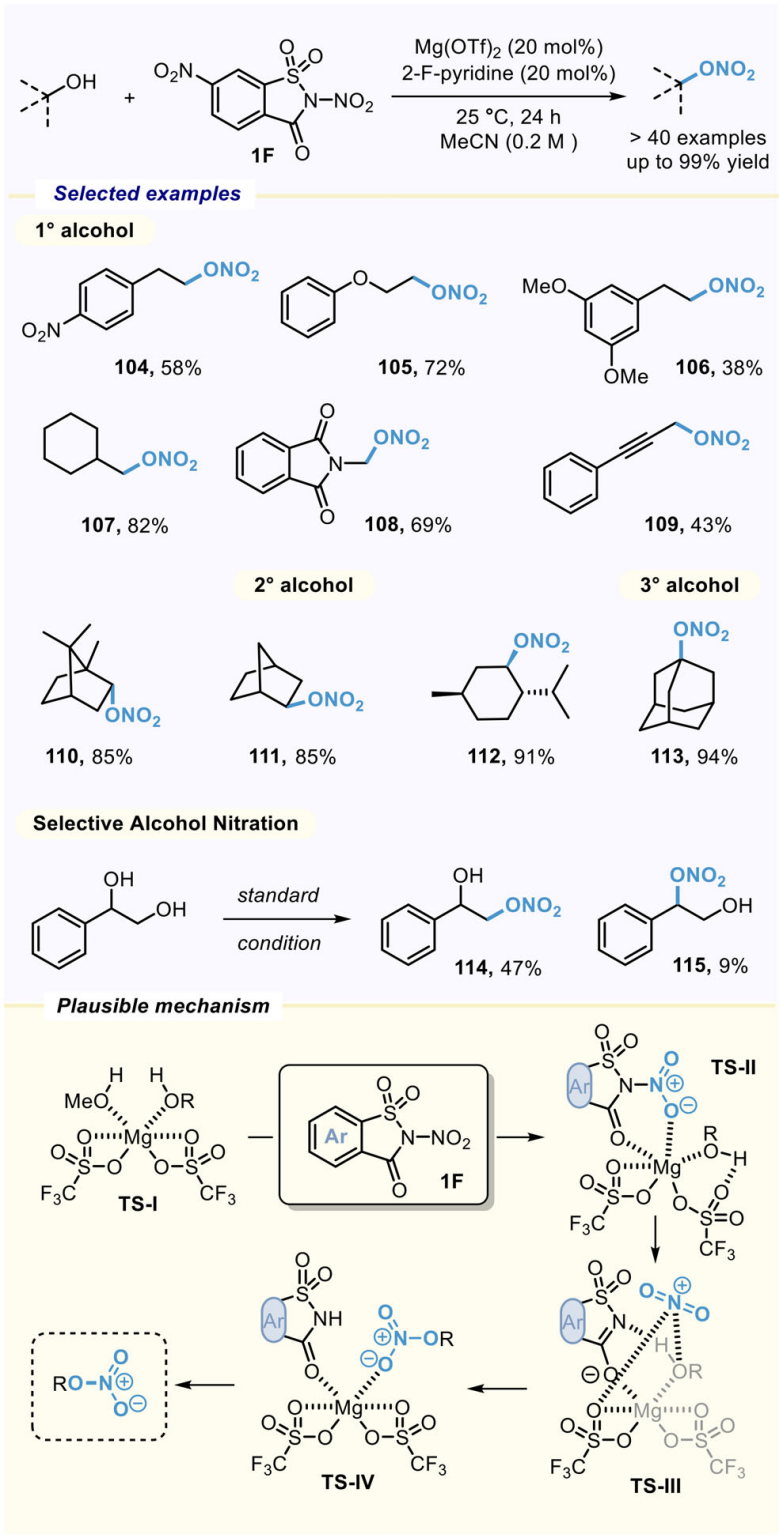
Catalytic O‐Nitration via Reagent Design (Nitronium Control).

The selectivity profile is also mechanistically informative. Competition experiments showed a clear preference for primary over secondary alcohol nitration, and this trend is reproduced in substrates containing both sites, where primary alcohol functionalization predominates. The method was further applied to the late‐stage nitration of complex molecules, including cholesterol, diosgenin, lavandulol, carbohydrate derivatives, and an indomethacin‐derived alcohol, demonstrating that the reagent design is compatible with multifunctional settings relevant to medicinal chemistry.

Mechanistically, the experimental optimization already points to a catalytic scenario distinct from traditional stoichiometric acid nitration. Mg(OTf)_2_ improves conversion substantially, whereas the addition of base suppresses competing aromatic nitration arising from background nitric acid release. The authors then used DFT calculations to probe the elementary steps in detail. These studies indicate a dual role for magnesium: it coordinates both the nitrating reagent and the alcohol substrate, thereby activating the *N*‐Nitrosaccharin toward cleavage while simultaneously increasing the acidity of the alcohol proton through Lewis's acid binding (**TS‐II**). The computed pathway supports a rate‐limiting *N–N* bond cleavage that generates a discrete yet strongly stabilized nitronium ion equivalent, followed by the formation of a short‐lived ROH⋯NO_2_^+^ complex (**TS‐III** to **TS‐IV**). From this species, proton transfer occurs essentially barrierlessly, collapsing directly to the nitrate ester product. Notably, this computational picture aligns closely with the classical Ingold formulation of alcohol nitration [62], but provides a modern molecular‐level rationale for achieving such selectivity under mild catalytic conditions. From a mechanistic perspective, the nitrating reagent is not simply a masked nitrate donor; instead, under magnesium activation, it becomes a controlled source of NO_2_^+^ character, while the alcohol is preorganized and activated within the same catalytic environment. This cooperative activation lowers the barrier for productive *O*‐nitration and helps explain the exceptional functional‐group tolerance observed experimentally. The authors also demonstrated flow‐scale‐up to the gram scale, highlighting the platform's practical value for the safer preparation of nitrate esters beyond small‐batch synthesis.

## 5 Decarboxylative Nitrooxylation

A decarboxylative approach to nitrate ester synthesis was reported by Li and coworkers, who developed a silver‐catalyzed nitrooxylation of aliphatic carboxylic acids using two complementary reaction manifolds (conditions A and B, Figure 11). Under condition A [AgNO_3_ (10 mol%), ligand **L1** (20 mol%), Na_2_S_2_O_8_ (2 equiv), NaH_2_PO_4_ (1.5 equiv) in DCE/H_2_O, 55°C, 12 h, Ar], a broad range of substrates undergo efficient conversion, whereas condition B [AgNO_3_ (20 mol%), ligand **L2** (20 mol%), CAN (4 equiv), NaHCO_3_ (3 equiv in DCE/H_2_O, 55°C, 12 h, Ar] provides a more strongly oxidizing environment suitable for less reactive or more functionalized systems [63]. From the substrate scope (Figure 11), both conditions enable the transformation of diverse aliphatic carboxylic acids into the corresponding nitrate esters. Simple substrates afford products **116** and **117** in good yields, while cyclic and/or functionalized systems bearing halides, ethers, and aryl groups are tolerated (**118–122**). Cyclic and sterically constrained frameworks also participate (**123**, **124**), as does di‐nitrooxylation of dicarboxylic acid chains (**125**), further demonstrating the breadth of the method. In general, condition A is more effective for less hindered substrates, whereas condition B is better suited to more challenging systems, reflecting differences in oxidant strength and radical‐generation efficiency.

**FIGURE 11:**
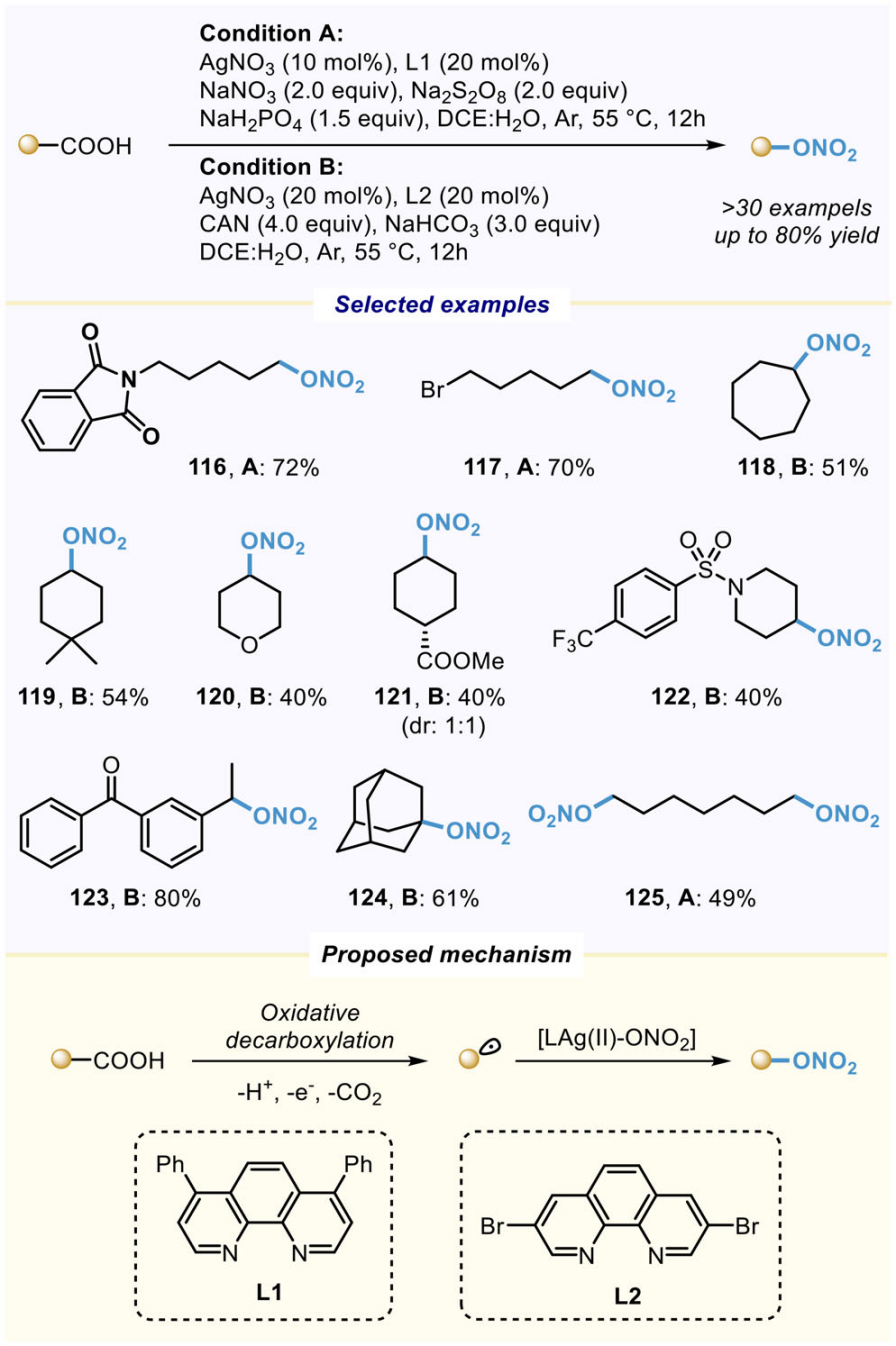
Silver‐catalyzed decarboxylative nitrooxylation of aliphatic carboxylic acids.

Mechanistically, both conditions proceed through a decarboxylative radical pathway. Single‐electron oxidation of the carboxylate generates an alkyl radical upon CO_2_ extrusion; under condition A, this is initiated by a sulfate radical anion (SO_4_•^−^) derived from persulfate, while under condition B, CAN directly mediates the oxidation. Concurrently, Ag(I) is oxidized to a higher‐valent species, plausibly an Ag(II)–ONO_2_ complex, which functions as the nitrate‐transfer agent. The nucleophilic alkyl radical is then trapped by this electrophilic nitrate species to furnish the C–ONO_2_ bond, followed by reduction to regenerate Ag(I).

Several control experiments reported in the study provide strong support for this mechanistic picture. The addition of radical scavengers (e.g., TEMPO or BHT) significantly suppresses product formation, indicating the involvement of freely diffusing carbon‐centered radicals. In addition, radical‐trapping experiments yield adducts consistent with alkyl radical intermediates. The necessity of both silver salt and external oxidants (persulfate or CAN) further supports a redox‐mediated catalytic cycle rather than a purely ionic nitrate transfer. Notably, the absence of AgNO_3_ leads to a dramatic reduction in conversion, highlighting its dual role as both a catalyst and a nitrate source. Together, these observations are consistent with a stepwise radical decarboxylation followed by polarity‐matched nitrate transfer from a high‐valent silver species.

## 6 NO_2_ / NO_3_ Radical Driven Nitrooxylation Pathways

Within radical NO_x_‐driven nitrooxylation chemistry, Taniguchi and co‐workers reported a seminal oxidative nitration of alkenes using *t*‐butyl nitrite and molecular oxygen, establishing a practical entry to *β*‐nitro alcohols and their corresponding nitrate esters under mild conditions [64]. From the substrate scope depicted in Figure 12, a range of terminal and internal alkenes undergo efficient difunctionalization, furnishing products **126–133** in moderate to good yields. Electron‐deficient alkenes preferentially deliver *β*‐nitro nitrate derivatives (**126–128**), with diastereoselectivity observed in certain cases (e.g., **128**, dr = 63:35), reflecting controlled radical addition. Unactivated aliphatic alkenes (**129–133**) are also competent substrates, consistent with less stabilized internal radical intermediates. Notably, no regioisomeric products were detected, indicating a highly selective radical addition of NO_2_• across the double bond.

**FIGURE 12:**
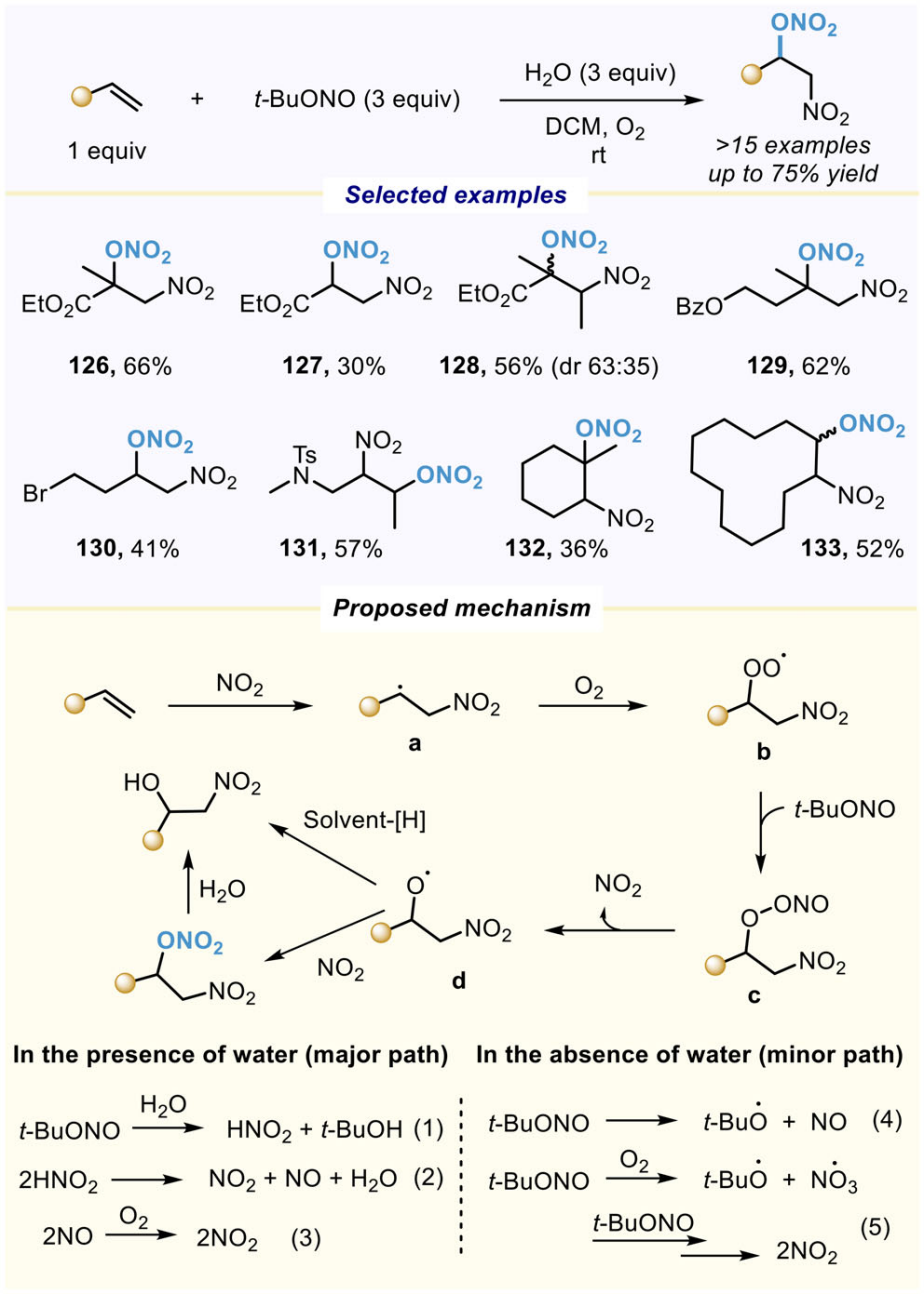
Oxidative nitration of alkenes with *t*‐butyl nitrite and oxygen.

Mechanistically (Figure 12), the transformation proceeds through a NO_x_‐mediated radical chain pathway. In the presence of water (major pathway), hydrolysis of *^t^* BuONO generates nitrous acid (HNO_2_), which decomposes to NO and NO_2_ (Equations 1–2). Subsequent oxidation of NO by O_2_ replenishes NO_2_ (Equation 3), establishing a steady‐state concentration of reactive nitrogen oxide species. Radical addition of NO_2_ to the alkene forms the carbon‐centered radical **a**, which is rapidly trapped by O_2_ to generate peroxy radical **b**. Reaction of **b** with *t*‐BuONO produces the peroxynitrate intermediate **c**, which undergoes O–O bond homolysis to give the alkoxy radical **d**. Radical recombination of **d** with NO_2_• furnishes the nitrate ester product.

Notably, two competing pathways govern NO_2_• generation: (i) HNO_2_ decomposition in the presence of water (major pathway), and (ii) direct homolysis or aerobic oxidation of *^t^* BuONO (minor pathway). From a mechanistic perspective, this system exemplifies a kinetically controlled radical cascade, wherein sequential NO_2_ addition, oxygen trapping, and peroxynitrate fragmentation collectively orchestrate C–NO_2_ and C–ONO_2_ bond formation. The dual formation of *β*‐nitro alcohols and nitrate esters thus reflects a delicate interplay between radical recombination and downstream hydrolytic processes.

In 2017, Punniyamurthy and co‐workers described a *t*‐BuONO/*N*‐hydroxylamine‐mediated protocol for direct C(sp^3^)–H nitrooxylation under aerobic conditions [65]. Here, PINO refers to the phthalimide *N*‐oxyl radical, while BtO is from HOBt (*N*‐hydroxybenzotriazole). From the substrate scope (Figure 13), the reaction shows good generality across styrenyl, aliphatic, and cyclic alkene substrates. A range of styrene substrates and *N*‑Hydroxylamines afford the corresponding nitrooxylated products **134–139**, with high diastereoselectivity observed in internal alkenes (e.g., **136**, dr = 18:1). Cyclic substrates (**137–140**) also participate, delivering products in 45–49% yield with good stereocontrol (*d.r*. up to 20:1), suggesting a controlled radical recombination step. In contrast, unactivated linear alkenes (**141–144**) give lower yields (28–32%). Mechanistically (Figure 13), the reaction is initiated by homolytic cleavage of *t*‐BuONO, generating *t‐*BuO• and NO•. Concurrently, the *N*‐hydroxylamine is oxidized to the corresponding aminoxyl radical (**a**). Radical addition to the alkene furnishes a secondary carbon‐centered radical **b**, which is intercepted by molecular oxygen to give the peroxy radical **c**. Subsequent interaction of **c** with *^t^* BuONO regenerates *t‐*BuO• and forms a peroxo intermediate **d**. Homolytic fragmentation of **d** produces the alkoxy radical **e**, which undergoes trapping by NO_2_• to deliver the nitrooxylated product. Control experiments reported in the paper support this radical manifold. The reaction is strongly dependent on the presence of oxygen, indicating its role in the interconversion of NO_x_ (NO• → NO_2_•). Radical inhibitors suppress product formation, supporting the involvement of carbon‐centered radical intermediates. Additionally, the requirement for both *t*‐BuONO and the *N*‐hydroxylamine indicates a dual radical system, in which aminoxyl radicals mediate HAT, and NO_x_ species enable downstream oxidation to the nitrate ester.

**FIGURE 13:**
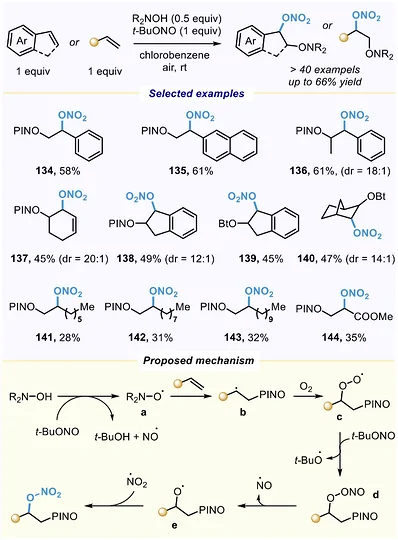
Aerobic nitrooxylation of alkenes with *t*‐butyl nitrite.

From a practical and mechanistic standpoint, *t*‐BuONO‐mediated alkene functionalization highlights the delicate balance between nitration (C–NO_2_ formation) and nitrooxylation (C–ONO_2_ formation), with the product selectivity largely dictated by the fate of the initially generated carbon‐centered radical intermediates. Studies from Maiti and coworkers demonstrated that *t*‐BuONO efficiently promotes direct olefin nitration under metal‐free conditions through NO_2_• radical addition pathways [66], ultimately furnishing nitroolefins via radical trapping and elimination processes. In contrast, the Taniguchi and Punniyamurthy systems operate under strongly aerobic conditions, where oxygen trapping of the intermediate carbon radicals generates peroxy/peroxynitrate intermediates that subsequently evolve into nitrate esters [63, 64]. Mechanistic studies in these nitrooxylation systems further revealed that molecular oxygen and water play crucial roles in promoting NO/NO_2_ interconversion and generating nitrate‐containing intermediates capable of delivering the ONO_2_ group. Collectively, these reports suggest that direct radical recombination with NO_2_• favors nitration, whereas efficient oxygenation and peroxynitrate formation shift the reaction manifold toward nitrooxylation.

## 7 Enantioselective SOMO Catalysis

In the context of enantioselective nitrooxylation, MacMillan and co‐workers introduced a conceptually distinct strategy based on SOMO catalysis, enabling asymmetric carbo‐oxidation of styrene to afford *γ*‐nitrate aldehydes with high stereocontrol [67]. This transformation is particularly notable in the evolution of nitrooxylation chemistry, as it departs from classical radical polarity‐driven addition and instead exploits a transient radical cation manifold to achieve enantio‐induction. The selected substrate scope, as summarized in Figure 14, demonstrates broad tolerance toward both aldehydes and styrene components. A variety of aldehydes bearing benzyl (e.g., **145**, 81%, 96% ee), cyclic, and functionalized substituents participate efficiently. In parallel, electronically diverse styrene, including electron‐rich and electron‐deficient aryl olefins, are competent coupling partners, affording products **146–150** in 88–94% yield and up to 97% ee.

**FIGURE 14:**
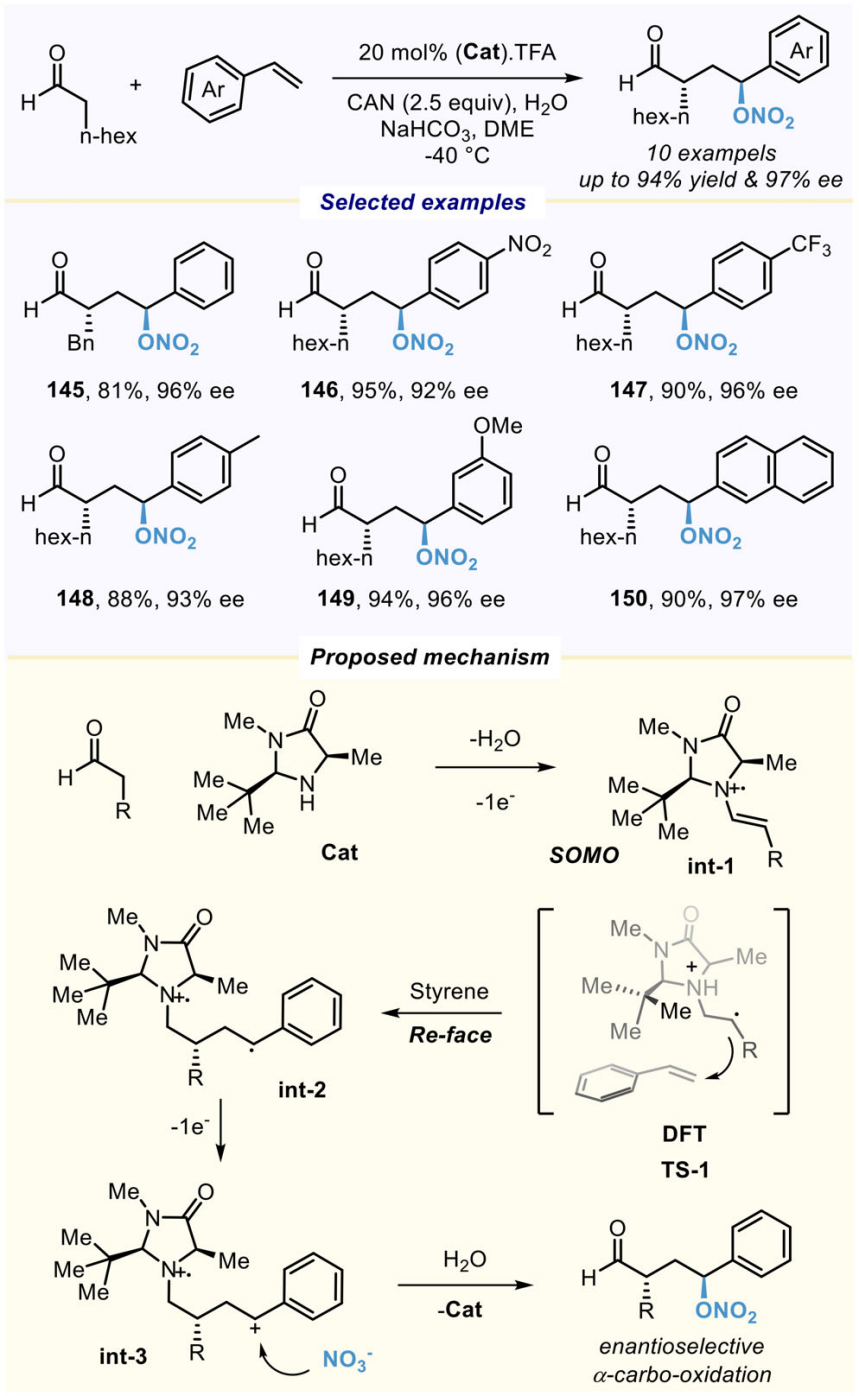
Enantioselective SOMO Catalysis for nitrooxylation of styrene.

Mechanistically, the reaction proceeds via a SOMO activation pathway initiated by condensation of the aldehyde with the chiral imidazolidinone catalyst to form an enamine intermediate, which undergoes single‐electron oxidation by ceric ammonium nitrate (CAN) to generate the corresponding radical cation (**int‐1**, SOMO‐activated species). From an orbital perspective, this 3π‐electron system exhibits a polarized SOMO that is sterically and electronically biased by the catalyst framework, enforcing a selective approach to the styrene from the *Re*‐face (**TS‐1**). Radical addition of the olefin yields benzylic radical intermediate **int‐2**, which is further oxidized to the corresponding carbocation (**int**‐**3**). At this stage, interception by nitrate anion, generated in situ from the reduction of Ce(IV), delivers the nitrooxylated product with concomitant formation of the second stereocenter. Hydrolysis of the iminium regenerates the catalyst and releases the *γ*‐nitrate aldehyde. Notably, DFT analysis supports a well‐defined transition state (**TS‐1**), in which steric shielding of the *Si*‐face by the *t*‐butyl substituent enforces enantioselective radical addition. This stereochemical model is consistent with the uniformly high enantioselectivities observed across the substrate scope. Moreover, the inability of the benzylic cation intermediate to undergo facile *β*‐elimination (unlike classical SOMO reactions) is a key mechanistic feature that enables productive trapping by nitrate, thereby diverting the pathway toward nitrooxylation rather than olefin formation.

## 8 C(sp^3^)–H Nitrooxylation

In 1987, Baciocchi and co‐workers reported the first example of photochemically induced nitrooxylation of inert C(sp^3^)–H bonds using ceric ammonium nitrate (CAN) in acetonitrile under remarkably mild conditions [68]. As summarized in the substrate scope (Figure 15), structurally rigid hydrocarbons such as adamantane, norbornane, and cyclohexane undergo direct functionalization to the corresponding nitrate esters, albeit with varying efficiencies. Adamantane and norbornane display high reactivity and selectivity (e.g., preferential tertiary C–H functionalization), whereas cyclohexane affords more modest yields (**151**, ∼30%), reflecting the higher bond dissociation energy and reduced radical stabilization of secondary C–H bonds.

**FIGURE 15:**
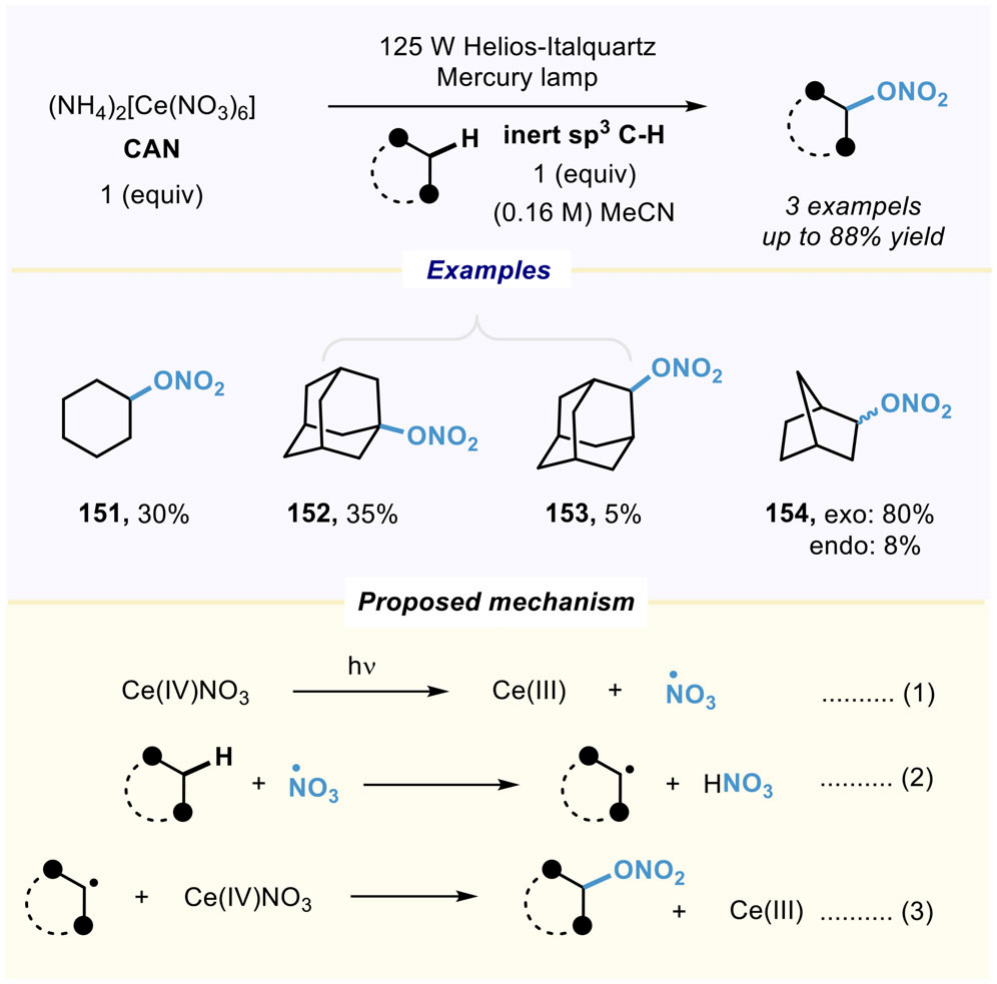
Photochemical oxidation and autooxidation of cycloalkanes.

Mechanistically, photolysis of CAN generates NO_3_• radicals via homolytic cleavage of the Ce–ONO_2_ bond (equation 1), a key step supported by earlier studies on alkylaromatic functionalization. These NO_3_• radicals serve as efficient hydrogen atom transfer (HAT) agents, abstracting hydrogen from the hydrocarbon substrate to generate an alkyl radical (eqution 2). The resulting carbon‐ centered radical is subsequently oxidized by Ce(IV) to form a cationic or tightly associated intermediate, which is then trapped by nitrate to deliver the nitrate ester product (equation 3), as illustrated in the proposed mechanism in Figure 15. In the presence of dioxygen, the system can operate catalytically, with O_2_ participating in autoxidation pathways that regenerate Ce(IV) from Ce(III) via peroxy radical intermediates, thereby sustaining the reaction cycle. Overall, this work established a foundational paradigm for nitrooxylation via radical HAT processes, in which NO_3_• serves as a key reactive intermediate, bridging classical nitrate chemistry with modern C–H functionalization strategies.

In a significant advance toward selective C–H nitrooxylation, Inoue and co‐workers (2011) reported a direct benzylic C(sp^3^)–H functionalization using a dual system comprising *N*‐hydroxyphthalimide (NHPI) as an organocatalyst and ceric ammonium nitrate (CAN) as both an oxidant and a nitrate source [69]. This strategy addresses a persistent challenge in benzylic oxidation chemistry: avoiding overoxidation to ketones or acids by installing a nitrooxy group as an electron‐withdrawing surrogate for alcohol functionality.

Based on the substrate scope (Figure 16), the method exhibits broad functional‐group tolerance across benzylic systems. A wide range of alkyl aromatics bearing diverse substituents on the side chain, including esters, carbamates, tosylates, nitriles, carboxylic acids, and halides, undergo smooth nitrooxylation to furnish the corresponding benzyl nitrates in generally moderate to excellent yields (e.g., **155–162**).

**FIGURE 16:**
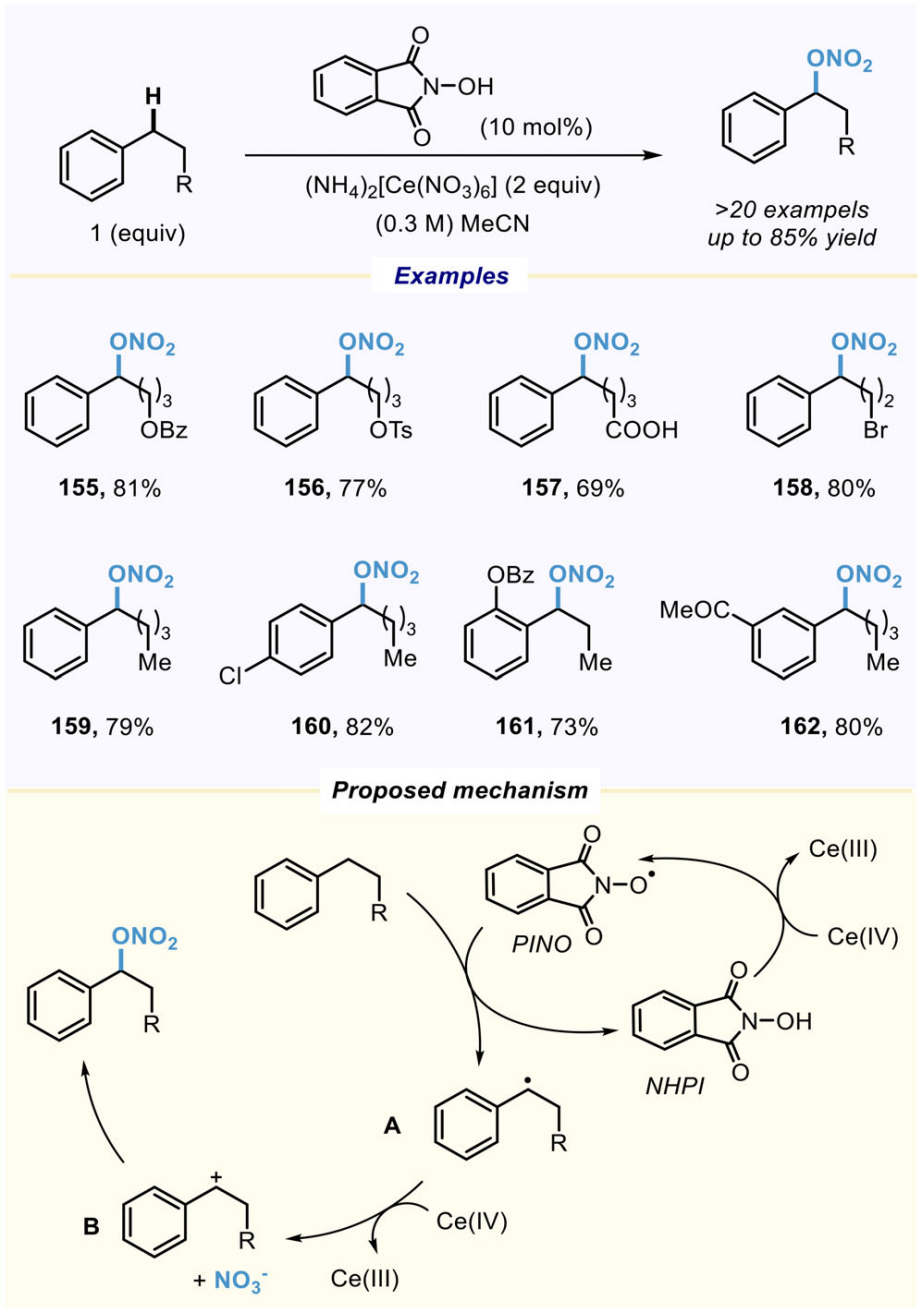
Direct oxidative installation of nitrooxy group at benzylic positions via C‐H activation.

Mechanistically, the transformation operates through a radical–polar crossover pathway, as outlined in the proposed cycle. Initial single‐electron oxidation of NHPI by Ce(IV) generates the phthalimide *N*‐oxyl radical (PINO), a well‐established HAT mediator. This species selectively abstracts a benzylic hydrogen atom, guided by the relatively low bond dissociation energy of benzylic C–H bonds, to generate a stabilized benzyl radical intermediate (**A**). Subsequent oxidation of the benzyl radical by Ce(IV) furnishes a benzyl cation (**B**), which is then trapped by nitrate anion to deliver the nitrate ester product. Several experimental observations support this mechanistic picture. The dramatic rate enhancement upon addition of catalytic NHPI (81% yield in 2 h vs 13% without NHPI over 24 h) indicates that HAT via PINO is the turnover‐limiting initiation step. The absence of significant ketone formation, even under strongly oxidizing conditions, suggests that the electron‐withdrawing ONO_2_ group suppresses further oxidation, consistent with a polarity inversion after functionalization. Moreover, the formation of minor acetamide byproducts (via solvent capture) implies the transient generation of a cationic intermediate, reinforcing the radical‐to‐cation crossover scenario. As an extension of directed C–H nitrooxylation chemistry, Kanai, Oisaki, and co‐workers (2016) developed a directing activator‐assisted aerobic nitrooxylation of unactivated secondary C(sp^3^)–H bonds, using an *N*‐hydroxyamide‐derived directing activator. In contrast to benzylic nitrooxylation, this strategy addresses the far more demanding problem of selectively oxygenating acyclic methylene C–H bonds. Under the optimized conditions, a combination of NaNO_2_, 2,6‐dichlorobenzoic acid, O_2_ (1 atm), and TFE at 40°C promotes oxidation with useful control over both site selectivity and oxidation state. As shown in the substrate scope in the figure, a range of DA‐tethered aliphatic alcohol derivatives undergo oxidation predominantly at the γ‐position, furnishing the corresponding nitrooxy products in preference to the overoxidized ketones (Figure 17) [70]. Simple butanol‐derived substrates (**163**‐**165**), as well as analogs bearing ester, phthalimide, nitrile, and acetate substituents (**166**‐**169**), are competent, generally yielding moderate yields of the nitrate ester products with only minor amounts of the corresponding ketones (**163’**‐**169’**). When the carbon chain is extended, oxidation can also occur at the *δ*‐position, although *γ*‐functionalization remains favored, with reported *γ/δ* ratios in the range of roughly 2.7:1 to 3.2:1. Notably, when the *γ*‐site is blocked by gem‐dimethyl substitution, the reaction switches cleanly to *δ*‐functionalization (**169**), albeit with some competing C–C bond cleavage. Mechanistically, the reaction relies on in situ‐generated NO_x_ chemistry coupled to a DA‐controlled radical relay. Optimization studies showed that neither nitrate salts nor acid alone is effective, whereas nitrite salt plus Brønsted acid is required, indicating that the active oxidizing/nitrosating species arise from the controlled generation of NO_x_ under aerobic conditions. The authors propose that nitrosonium ion (NO^+^) is generated from NaNO_2_ and the acid, and that this species oxidizes the *N*‐hydroxyamide moiety to the corresponding amidoxyl radical. Because this radical is tethered to the substrate, it undergoes selective 1,5‐HAT or 1,6‐HAT to abstract a *γ*‐ or *δ*‐hydrogen atom, generating a carbon‐centered radical at a defined remote site. Molecular oxygen then traps this radical to give a peroxyl radical intermediate, which is subsequently intercepted by NO. Rearrangement and homolysis of the resulting adduct furnish the nitrate ester product (path a in the proposed mechanism), whereas competing abstraction at the α‐oxy position by NO_2_, either directly or via regeneration of the amidoxyl radical, leads to the ketone byproduct (path b/b′).

**FIGURE 17:**
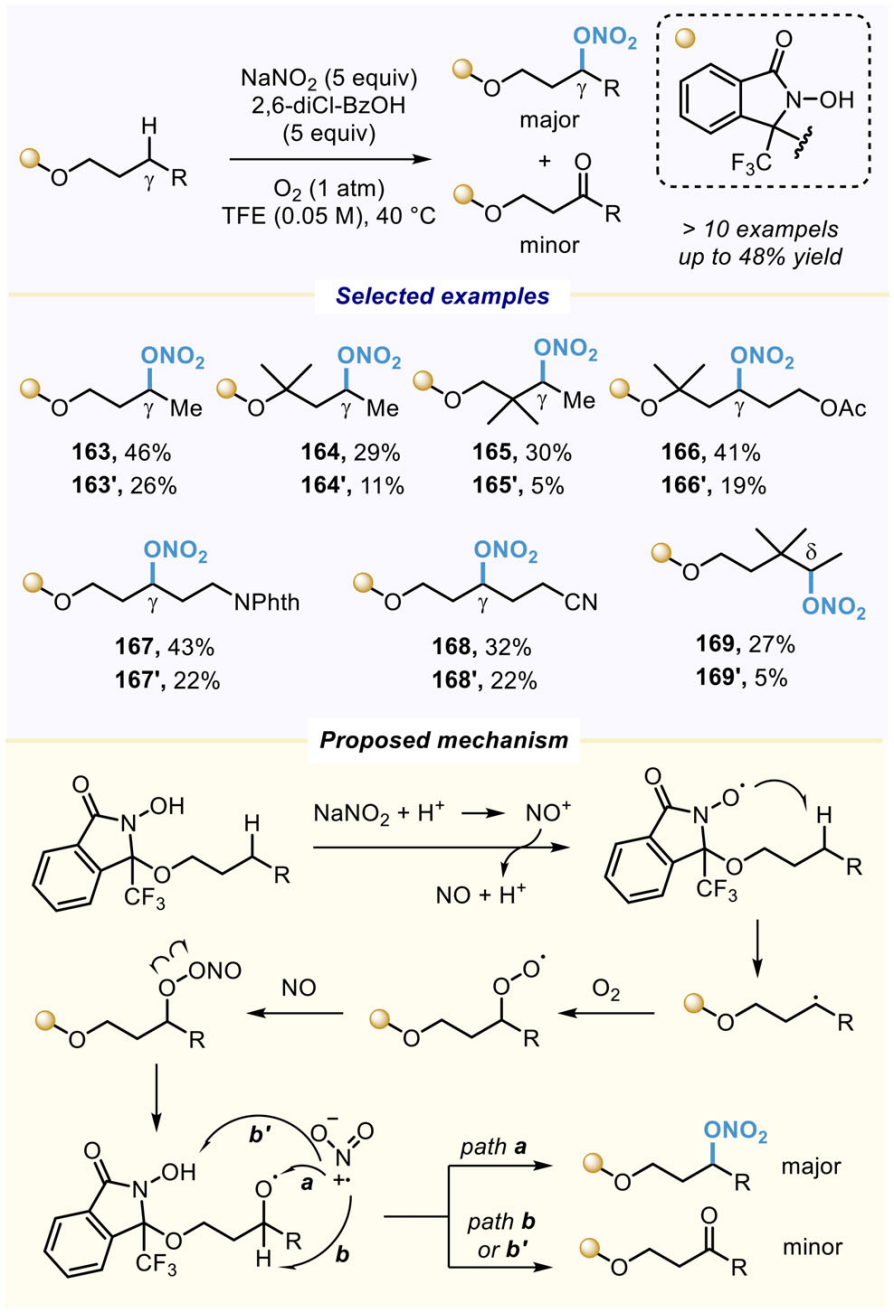
Chemo‐ and regioselective intramolecular C(sp^3^)–H activation via N‐hydroxyamide directing activators (DA).

Several observations support this mechanistic picture. First, the requirement for both nitrite and Brønsted acid points to a NO_x_‐mediated radical initiation step rather than simple nitrate transfer. Second, the strong *γ*‐selectivity and switch to *δ*‐oxidation when the *γ*‐site is blocked are fully consistent with a tether‐controlled intramolecular HAT manifold rather than nondirected oxidation. Third, the formation of nitrate esters rather than direct alcohols is mechanistically significant: the ONO_2_ group acts as a built‐in protecting group, suppressing further oxidation of the initially formed C–O bond and thereby solving a central selectivity problem in methylene oxygenation. This oxidation‐state control is one of the most important features of the method.

In a conceptual advance that merges directing‐group‐assisted C–H activation with nitrate‐transfer chemistry, palladium‐catalyzed nitrooxylation of unactivated C(sp^3^)–H bonds has emerged as a powerful strategy for the site‐selective installation of the –ONO_2_ motif under relatively mild conditions. Central to these approaches is the use of bidentate *N*,*N*‐chelating auxiliaries, which enforce proximity‐driven activation via cyclopalladation, thereby overcoming the inherent inertness and low site discrimination of aliphatic C–H bonds (Figure 18).

**FIGURE 18:**
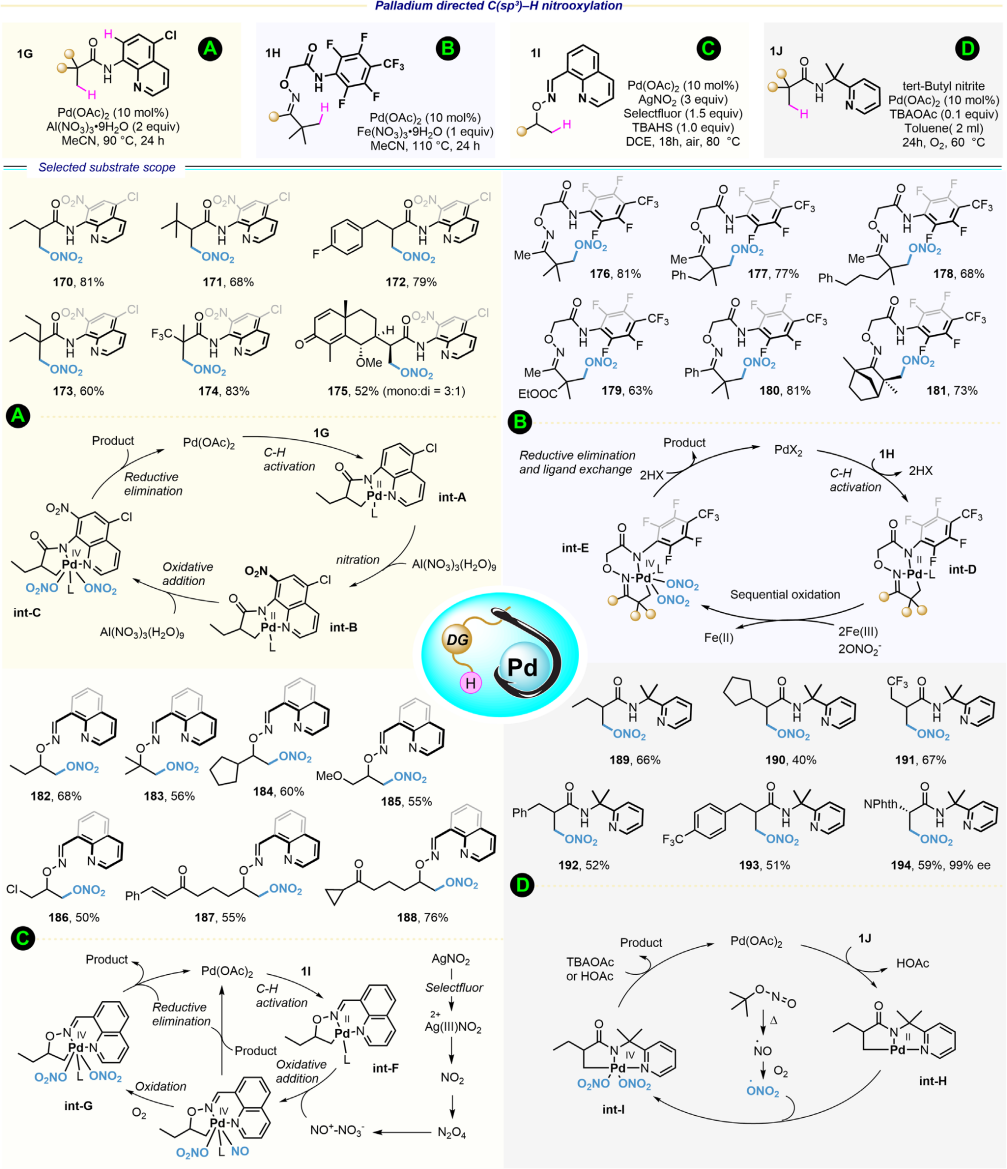
Palladium‐catalyzed selective C(sp^3^)−H nitrooxylation.

A representative implementation by Luo, Jiang, and co‐workers employs a modified quinoline auxiliary in combination with Al(NO_3_)_3_·9H_2_O, which serves as both nitrate source and terminal oxidant [71]. Notably, in situ nitration of the 5‐chloro‐8‐aminoquinoline auxiliary (**1G**) appears to enhance its directing ability and facilitate auxiliary cleavage. The method exhibits broad substrate generality across α‐branched aliphatic amides, delivering *γ*‐C–H nitrooxylated products in moderate to good yields (60–83%). Substrates bearing electron‐withdrawing groups (e.g., CF_3_) or distal aryl substituents are well tolerated, indicating that the C–H activation step is governed predominantly by geometric rather than electronic factors. More complex scaffolds, however, exhibit diminished selectivity, reflecting competition among multiple accessible C–H sites within the palladium coordination sphere (e.g., **170–175**, Figure 18A).

Mechanistically, this transformation proceeds via a classical Pd(II)/Pd(IV) manifold. Following coordination and CMD‐type C–H activation to form a cyclopalladated intermediate (**int‐A**), nitrate coordination and oxidation generate a high‐valent Pd(IV) species (**int‐B** to **int‐C**). From an orbital perspective, the strong σ‐donating environment of the bidentate auxiliary stabilizes this high‐valent intermediate, lowering the barrier for oxidative addition. Subsequent reductive elimination forges the C–ONO_2_ bond. The absence of radical‐derived byproducts supports an inner‐sphere, polarity‐controlled pathway.

A complementary study by Yu, Jiang, and co‐workers extends this paradigm to ketone and amide substrates using aminooxyamide directing groups (e.g., **176–181**) (Figure 18B) [72]. The reaction displays broad scope across aliphatic methyl ketones and aryl‐substituted systems, maintaining high *β*‐site selectivity even in the presence of competing enolizable positions. Notably, palladacycle intermediates (**int‐D**) were isolated and shown to be catalytically competent, providing direct structural evidence for the involvement of chelation‐controlled intermediates. Mechanistically, the reaction proceeds through sequential Fe(III)‐ mediated oxidation events, enabling access to a Pd(IV) species (**int‐E**), from which reductive elimination delivers the nitrate ester. The dual role of Fe(NO_3_)_3_·9H_2_O as both oxidant and nitrate donor highlights an efficient redox‐coupled transfer process. For more weakly coordinating substrates, such as native amides, a modified catalytic system is required. Incorporation of a pyridine‐3‐sulfonic acid ligand, together with AgNO_3_ as nitrate source and N‐iodosuccinimide (NIS) as a bystanding oxidant in HFIP, enables efficient *β*‐C(sp^3^)–H nitrooxylation [72]. The requirement for NIS reflects a mechanistic shift toward a more classical two‐electron oxidation manifold, facilitating access to high‐valent Pd intermediates in the absence of strongly chelating auxiliaries.

Extending the directing‐group concept further, Jiang and co‐workers demonstrated that exo‐oxime auxiliaries can enable *β*‐C(sp^3^)–H nitrooxylation of masked alcohols. Under Pd(OAc)_2_/AgNO_2_/Selectfluor conditions, a wide range of secondary and tertiary substrates undergo selective functionalization with good β‐site selectivity and broad functional group tolerance (e.g., **182–188**) (Figure 18C) [73]. Mechanistically, this system illustrates a hybrid radical–polar crossover regime: Selectfluor‐mediated oxidation of Ag(I) generates NO_2_ radicals, which ultimately participate in oxidation of the Pd(II) palladacycle (**int‐F**) to a Pd(IV) intermediate (**int‐G**). Despite the involvement of radical species, C–ONO_2_ bond formation proceeds through reductive elimination from a high‐valent organometallic intermediate.

A distinct mechanistic variant was reported by Shi and co‐workers, employing *t*‐butyl nitrite (TBN) and molecular oxygen (Figure 18D) [74]. This system is notable for its operational simplicity and use of O_2_ as a green oxidant. The reaction accommodates a wide range of aliphatic carboxamides, including sterically demanding substrates, and preserves stereochemical integrity in amino acid derivatives (e.g., **189–194**) (up to 99% ee). Mechanistic interrogation reveals a more pronounced radical character: a measurable kinetic isotope effect (KIE ≈ 2.3) implicates C–H cleavage in the rate‐determining step, while radical scavengers suppress reactivity. Furthermore, ^1^ ^8^O_2_‐labeling confirms direct incorporation of oxygen from O_2_ into the nitrate ester. From a mechanistic standpoint, thermal homolysis of TBN generates NO•, which is oxidized by O_2_ to reactive nitrogen oxide species that oxidize the Pd(II) intermediate (**int‐H**) to Pd(IV) (**int‐I**), followed by reductive elimination. This system, therefore, exemplifies a tightly coupled radical–organometallic pathway.

Taken together, these studies delineate a coherent mechanistic landscape for Pd‐catalyzed C(sp^3^)–H nitrooxylation. Despite variations in reagent systems, a unifying feature is the formation of a cyclopalladated intermediate, followed by oxidation to a high‐valent Pd species and reductive elimination to form the C–ONO_2_ bond. The primary divergence lies in how this oxidation step is achieved: purely ionic pathways (e.g., nitrate‐assisted Pd(II)/Pd(IV) cycles) contrast with hybrid radical–polar crossover systems in which nitrogen oxide radicals serve as oxidants. From a design perspective, directing‐group architecture remains critical, not only for site selectivity but also for stabilizing high‐valent intermediates and modulating reaction kinetics.

Nevertheless, limitations persist, including the need for auxiliary installation/removal, reliance on stoichiometric oxidants or metal nitrates, and diminished efficiency for sterically congested substrates. Future advances will likely focus on transient directing groups, catalyst‐controlled selectivity, and integration with photochemical or electrochemical platforms to access more sustainable and broadly applicable nitrooxylation manifolds.

## 9 Photocatalytic Nitrooxylation

In the context of photoredox‐enabled nitrooxylation, Katayev and co‐workers disclosed a conceptually distinct strategy based on a hypervalent iodine–derived nitrooxylating reagent, which is activated under visible‐light photoredox conditions to enable direct C–H functionalization of heterocycles (Figure 19) [56].

**FIGURE 19:**
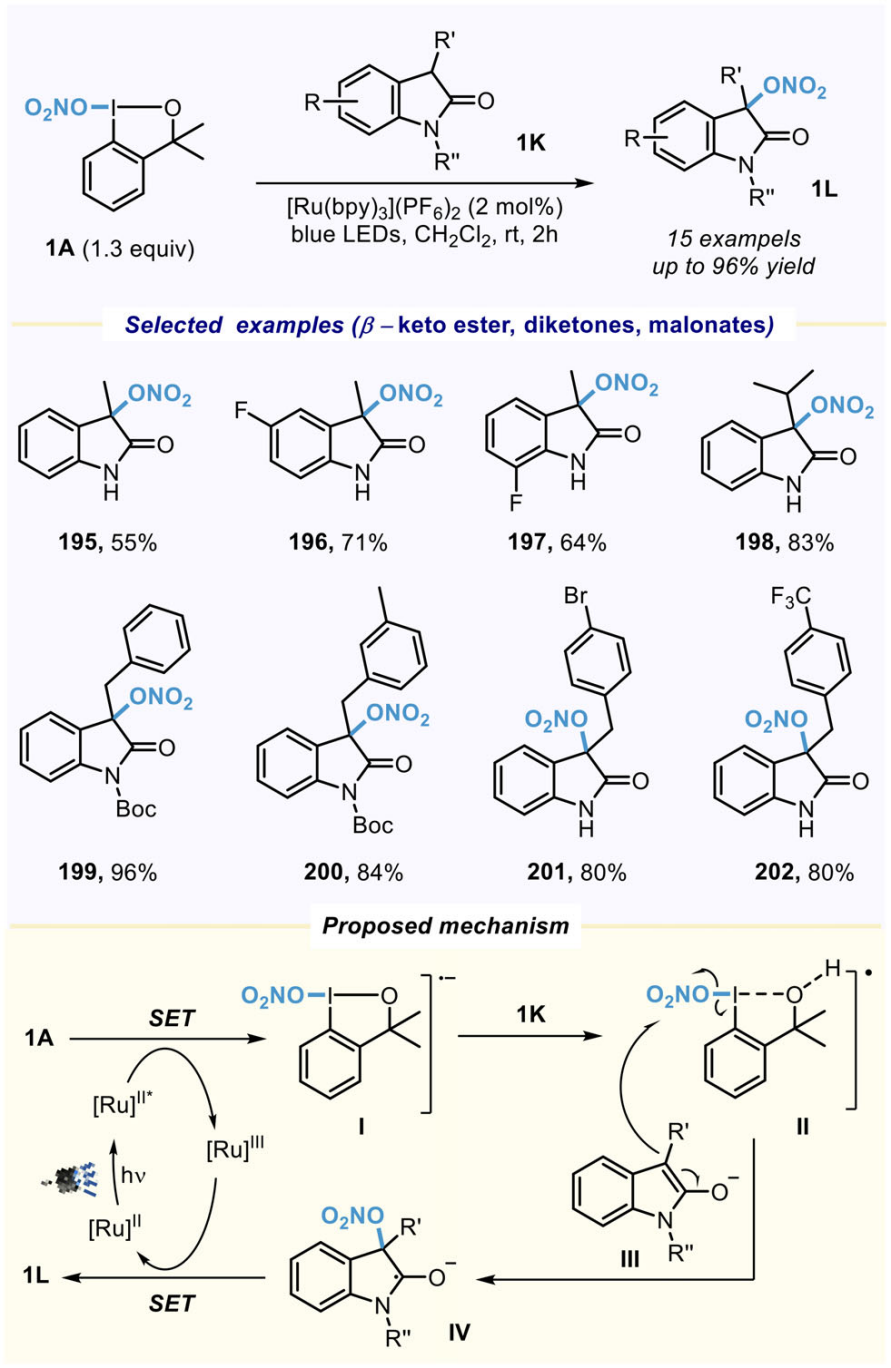
Photocatalytic hypervalent‐iodine‐based activation for nitrooxylation reaction.

From the selected substrate scope illustrated in Figure 19, a range of oxindole derivatives undergo efficient C3‐nitrooxylation to deliver products (**195–202**) in generally moderate to good yields. Both electron‐donating and electron‐withdrawing substituents on the aromatic ring are well tolerated (e.g., halides, CF_3_, and ester groups), variation at the C3 position is also accommodated, including alkyl and benzyl substituents, and different N‐protecting groups such as Boc, Cbz, and acetyl are compatible, highlighting the chemoselectivity of the system. The consistently high regioselectivity for C3‐functionalization reflects the inherent acidity of the oxindole *α*‐position and the controlled generation of a nucleophilic enolate intermediate under the reaction conditions. Mechanistically, this transformation proceeds through a photoredox SET manifold rather than a classical nitrate radical pathway. Upon irradiation with blue LEDs, the excited photocatalyst [Ru(bpy)_3_]^2^^+^ undergoes oxidative quenching by the hypervalent iodine reagent 1A, generating the radical anion I (as supported by Stern–Volmer quenching studies). The formation of I is experimentally substantiated by pulse radiolysis, which reveals a transient absorption at ∼420 nm corresponding to the reduced iodine species. Notably, this observation is mechanistically significant because it argues against immediate homolytic fragmentation to a free NO_3_• radical. Instead, **I** act as a redox‐active base, deprotonating the oxindole substrate **1K** to generate the oxo intermediate **II** and the enolate intermediate **III**, which evolves to **IV** through a concerted nitrooxy transfer. Subsequent oxidation of **IV** closes the catalytic cycle, regenerating the ground‐state photocatalyst. A notable mechanistic insight from this study is that nitrooxy transfer occurs without the intermediacy of a freely diffusing NO_3_• radical. Instead, the reaction proceeds via an inner‐sphere or concerted outer‐sphere pathway involving the reduced iodine reagent. This distinction is critical, as it rationalizes the high chemoselectivity and suppression of overoxidation or dinitrooxylation typically associated with NO_x_ radical chemistry. Furthermore, control experiments confirm the necessity of both light and photocatalyst, while UV–Vis spectroelectrochemical studies support the role of **I** as a sufficiently basic species to deprotonate the substrate.

Along similar nonphotochemical lines, You and coworkers developed a catalyst‐free nitrooxylative dearomatization of *β*‐naphthols using hypervalent iodine reagents, enabling direct formation of nitrooxy‐substituted naphthalenones under mild conditions. The process proceeds via electrophilic activation of the aromatic π‐system followed by C–ONO_2_ bond formation and rearomatization, delivering products in good to excellent yields (up to ∼87%) with high chemoselectivity [75].

In a complementary direction, a catalyst‐free variant has recently been disclosed by the same group, wherein hypervalent iodine‐mediated nitrooxylation of indoles is followed by in situ nucleophilic substitution, enabling rapid access to 3‐substituted oxindoles. Mechanistically, the initially formed nitrooxylated intermediate serves as a reactive electrophilic platform that undergoes substitution with external nucleophiles (e.g., alcohols), thereby effecting overall dearomatization under mild, metal‐free conditions [76].

In continuation of photocatalyzed nitrooxylation strategies, Ritter and co‐workers introduce an organophotocatalytic system that enables direct alkene nitrooxylation under oxidative conditions, using concentrated nitric acid as both the nitrate and hydrogen sources (Figure 20) [77]. The transformation proceeds under blue LED irradiation in the presence of an acridinium‐type photocatalyst (**1** **M**), affording anti‐Markovnikov nitrate esters.

**FIGURE 20:**
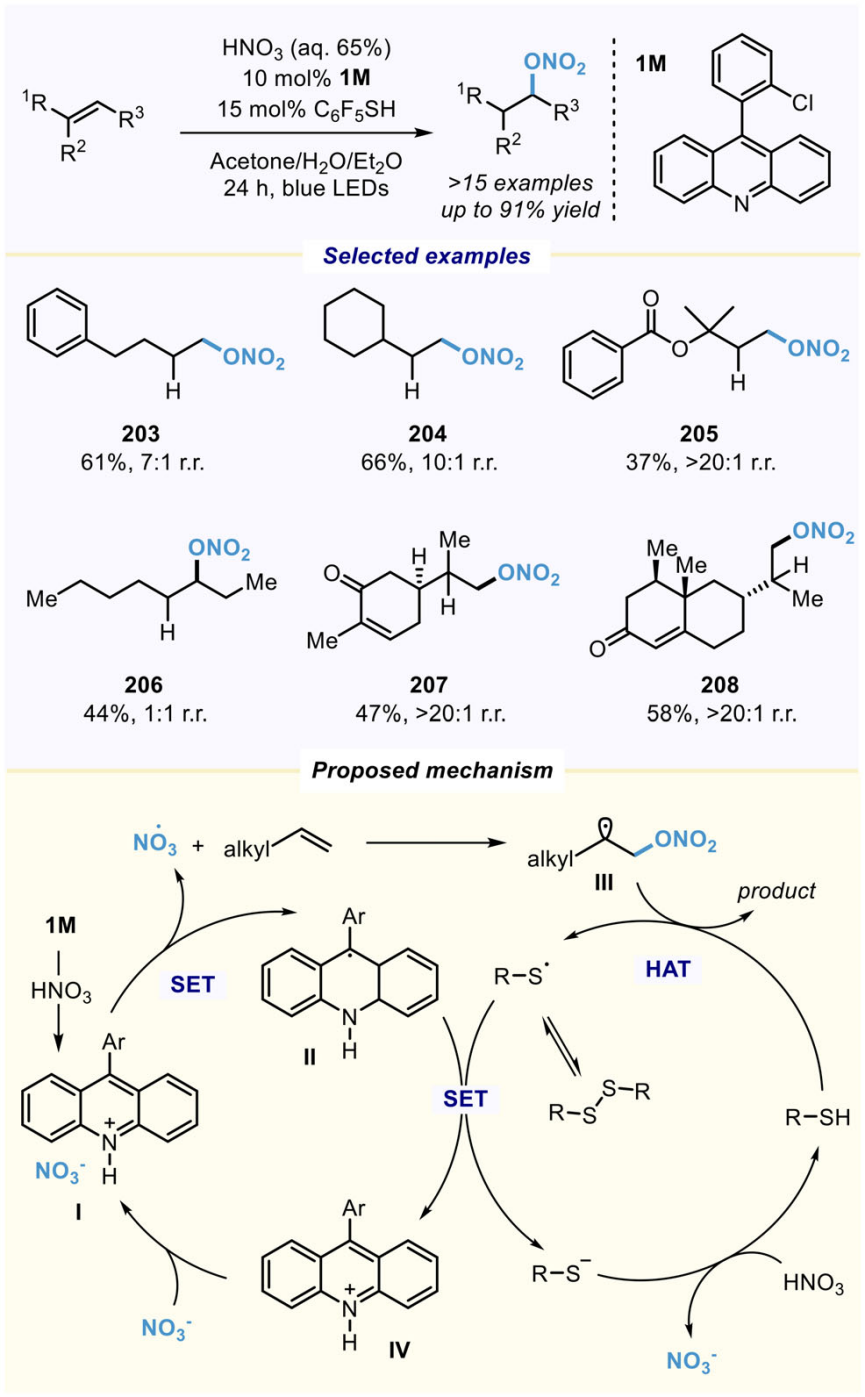
Anti‐Markovnikov hydro‐nitrooxylation of olefins via visible‐light photocatalysis.

Within the selected substrate scope, a range of unactivated alkenes is competent, including linear terminal alkenes, cyclic systems such as cyclohexene derivatives, ester‐containing alkenes, and more highly substituted aliphatic frameworks, all of which undergo nitrooxylation to afford the corresponding nitrate esters in moderate yields (**203–205**). Notably, substrates bearing proximal heteroatoms or activated C–H bonds are also well tolerated, furnishing the desired products without significant overoxidation (e.g., **206–208**), thereby highlighting the broad functional group compatibility. Mechanistically, the reaction proceeds via a photoredox‐mediated radical pathway initiated by excitation of the organic photocatalyst (**1M**→ **1M** ^⁎^), which subsequently oxidizes nitrate anion (NO_3_^−^) (from intermediate **I**) through a single‐electron transfer (SET) event to generate the electrophilic NO_3_• radical. The NO_3_• radical then adds across the alkene to furnish a carbon‐centered radical intermediate (**III**), consistent with anti‐Markovnikov regioselectivity dictated by radical stability. Subsequent hydrogen atom transfer (HAT) from a suitable donor (R–SH) converts intermediate **III** into the corresponding nitrate ester product. In parallel, the oxidized photocatalyst (intermediate **II**) accepts one electron from a suitable electron donor (R–S^−^), regenerating the ground‐state catalyst (**IV**) and closing the catalytic cycle by forming an ion pair with the nitrate ion. Mechanistic studies support this radical pathway: (i) inhibition in the presence of radical scavengers confirms the involvement of radical intermediates; (ii) the necessity of light and photocatalyst establishes a photoredox‐driven SET manifold.

## 10 Electrochemical Approaches

As an early entry into electrochemical nitrooxylation, Shono, Kashimura, and co‐workers demonstrated that anodic oxidation of nitrate salts generates NO_3_• radicals that can directly add to alkenes [78]. Simple terminal alkenes such as oct‐1‐ene undergo nitrooxylation in moderate yield (up to ∼74%) under mixed‐solvent conditions, whereas related linear and cyclic alkenes typically afford the corresponding nitrate esters in the ∼50–78% range. More substituted alkenes exhibit altered reactivity; 1,1‐disubstituted systems preferentially undergo intramolecular trapping to give heterocyclic products in ∼69–77% yield, whereas internal alkenes such as oct‐2‐ene provide mixtures of regioisomeric nitrate esters. Overall, the scope remains narrow, focusing on simple alkene classes with limited functional‐group tolerance. Mechanistically, anodic oxidation of NO_3_^−^ produces the electrophilic NO_3_• radical, which adds to the alkene to generate a carbon‐centered radical intermediate. Subsequent hydrogen‐ atom transfer from the solvent (MeCN/H_2_O or MeCN/H_2_O/Et_2_O) delivers the product.

Building on these early anodic NO_3_•‐mediated systems, Frontana‐Uribe, Piscil, and co‐workers reported a conceptually distinct electrochemical nitrooxylation of 3‐alkoxyamino lactams, in which substrate oxidation initiates the transformation rather than direct nitrate oxidation (Figure 21) [79]. Under electrochemical conditions (graphite electrodes, MeCN, nitrate electrolyte), a range of cyclic lactams undergo efficient *α*‐functionalization, furnishing nitrate esters (**209–216**) in moderate yields. The scope, although structurally focused, demonstrates tolerance for ring‐size variation and the incorporation of substituents, including several other functional groups. Mechanistically, this transformation departs from the classical NO_3_• addition pathway. Anodic oxidation of the 3‐alkoxyamino lactam generates a radical cation, which subsequently evolves into a reactive species that is trapped by nitrate to furnish the product.

**FIGURE 21:**
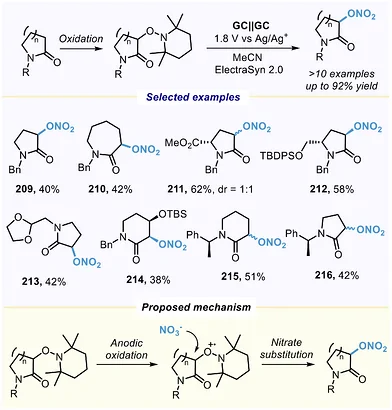
Electrochemical nitrooxylation of 3‐alkoxyamino lactams.

## 11 Mechanochemical Approaches

Mechanochemistry has emerged as a compelling platform for sustainable bond formation, particularly in nitrooxylation, where traditional protocols often rely on highly acidic and oxidizing media. From a reactivity standpoint, the key advantage lies in the direct conversion of mechanical energy into chemical activation, enabling electrophilic transformations under solvent‐minimized conditions while suppressing competitive overoxidation pathways. In this context, Katayev and co‐workers reported a mechanochemical nitration strategy based on a bench‐stable saccharin‐ derived reagent (**1F**), operating under liquid‐assisted grinding (LAG) conditions in the presence of catalytic Sc(OTf)_3_. This protocol circumvents the need for classical mixed‐acid systems by leveraging controlled nitronium transfer under mild conditions (Figure 22) [80].

**FIGURE 22:**
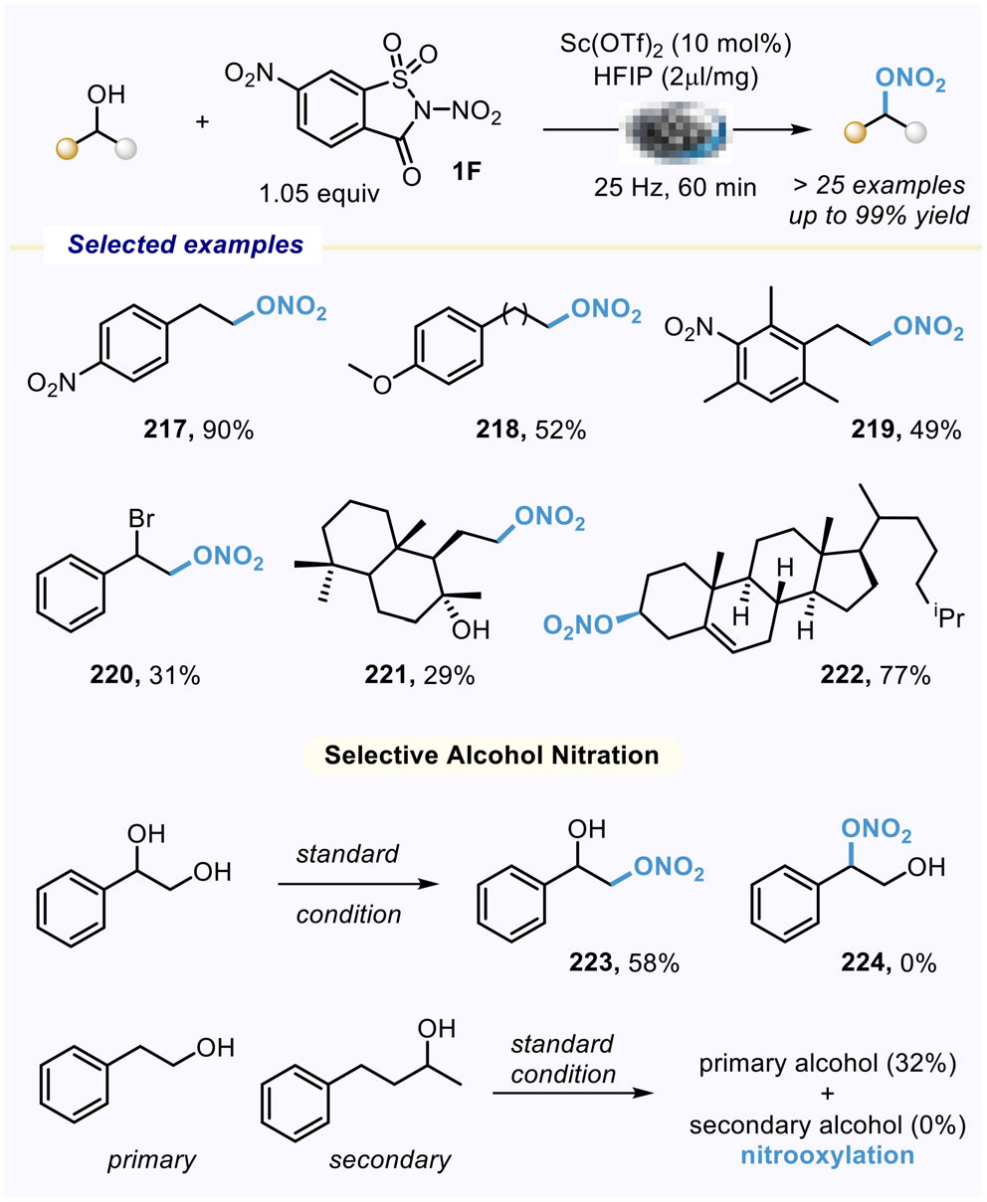
Mechanochemical nitration of alcohols using bench‐stable organic nitrating reagent.

Based on the substrate scope illustrated in Figure 22, the methodology demonstrates broad applicability to alcohol *O*‐nitration. Primary alcohols are converted efficiently to the corresponding nitrate esters, while structurally diverse substrates, including ether, sterically encumbered, and halogenated substrates (**217**‐**220**), are accommodated with moderate to good efficiency. Notably, complex and functionalized molecules, including polycyclic scaffolds (**221** & **222**), also undergo productive nitrooxylation. A salient feature emerging from the selectivity studies is the pronounced preference for primary over secondary alcohol nitration, as evidenced by selective formation of **223** (58%) with no detectable overfunctionalization (**224**, 0%), consistent with a kinetically controlled electrophilic substitution at the less hindered site.

Mechanistically, although no radical intermediates are implicated, the reaction proceeds through Lewis's acid activation of the nitrating reagent, generating a nitronium‐like electrophile that engages the alcohol oxygen. From an orbital perspective, coordination of Sc(OTf)_3_ enhances polarization of the N–NO_2_ bond within the reagent, effectively lowering the barrier to NO_2_^+^ transfer as discussed before in Figure 10. Collectively, this work highlights how mechanochemical activation can be harnessed to achieve controlled nitrooxylation under operationally simple, environmentally benign conditions while maintaining high selectivity.

In continuation of Katayev's work, this mechanochemical approach provides an alternative entry to alkene nitrooxylation under solvent‐free conditions, using Fe(NO_3_)_3_ and TEMPO under ball milling [81]. From the selected substrate scope (Figure 23), styrenyl substrates bearing both electron‐donating and electron‐withdrawing substituents (**225–230**) undergo efficient difunctionalization, generally affording the desired products in good to high yields. Also, unactivated aliphatic alkenes (**231–233**) participate to afford the desired nitro‐nitrooxylated di‐functionalized product. Overall, the method shows reasonable functional‐group tolerance and is not limited to activated olefins.

**FIGURE 23:**
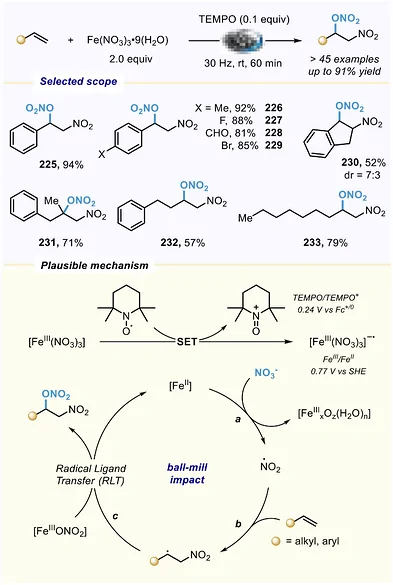
Mechanochemistry drives alkene difunctionalization via radical ligand transfer and electron catalysis.

Mechanistically (Figure 23), the reaction proceeds through a radical pathway involving coupled electron transfer and ligand transfer steps. An initial single‐electron transfer between Fe(NO_3_)_3_ and TEMPO generates a TEMPO^+^/Fe(II) pair, establishing a redox cycle. Under ball‐milling conditions, the Fe–ONO_2_ species can undergo homolysis to generate NO_2_• (step **a**), which adds to the alkene to form a carbon‐centered radical intermediate (step **b**). This intermediate is then trapped via radical ligand transfer (RLT) from an iron–nitrate complex, delivering the nitrate ester product (step **c**). The role of mechanical energy is to promote bond homolysis and sustain the radical process in the absence of solvent or light.

The mechanistic studies reported in the paper support this interpretation. The involvement of the TEMPO/TEMPO^+^ redox couple together with the Fe(III)/Fe(II) cycle is consistent with an electron‐catalytic process. The identification of NO_2_• as a key reactive intermediate, along with the requirement for ball‐milling, supports a radical pathway rather than an ionic mechanism. Taken together, this study shows that mechanochemistry can be used to access nitrooxylation via radical ligand transfer under mild, solvent‐free conditions. It provides a complementary approach to photochemical and electrochemical methods, where the mode of energy input controls the generation of reactive NO_x_ species and the overall reaction kinetics.

## 12 Limitations, Safety, and Practical Considerations

Despite the increasing sophistication of nitrooxylation strategies, the practical implementation of these reactions remains intrinsically constrained by the physicochemical properties of the nitrate ester functionality itself. In line with long‐standing observations in O‐nitration chemistry, nitrate esters are inherently unstable, often prone to rapid decomposition under thermal or acidic conditions, and, in extreme cases, can behave as energetic materials with explosive character, particularly when low‐molecular‐weight or densely functionalized frameworks are involved. Mechanistically, this instability can be traced to the weak, highly polar O–N bond, which undergoes facile homolysis to generate alkoxy and NO_2_‐derived radical species, thereby initiating uncontrolled chain processes. As a consequence, even well‐defined transformations must be conducted under carefully moderated conditions, where temperature control and avoidance of accumulation become critical. These features directly translate into scalability limitations, where exothermicity and radical‐propagation kinetics are amplified, often leading to poor reproducibility in batch systems. Continuous‐flow platforms partially alleviate these concerns by improving heat dissipation and reagent dosing, yet they introduce their own operational complexity and demand precise control over residence time and mass transport. In parallel, the involvement of NO_x_ species, either as reagents or transient intermediates, poses an additional layer of difficulty, given their toxicity, corrosive nature, and dynamic speciation equilibria that can divert productive pathways toward over‐oxidation or competitive nitration. From a process standpoint, efficient containment and quenching of NO_x_ remain essential but nontrivial. Finally, although recent catalytic approaches have reduced reliance on harsh mixed‐acid systems, many protocols still require stoichiometric nitrate sources or generate significant inorganic waste, and catalyst deactivation under NO_x_‐rich environments is insufficiently understood, thereby limiting both turnover efficiency and long‐term sustainability.

## 13 Outlook and Future Directions

In this context, the future development of nitrooxylation chemistry will likely depend less on identifying new reagent classes and more on achieving finer mechanistic control over nitrate‐transfer processes. A particularly underexplored dimension is enantioselective nitrooxylation, in which the central challenge is controlling the stereochemical outcome of inherently fast radical recombination events. From a kinetic perspective, the diffusion‐controlled capture of carbon‐centered radicals by NO_2_ or related species leaves little opportunity for asymmetric induction, suggesting that effective solutions will require preorganization, either through chiral ion‐pairing, constrained radical cages, or metal‐mediated inner‐sphere pathways that impose spatial control during bond formation. Similarly, the selective nitrooxylation of unactivated aliphatic C(sp^3^)–H bonds remains an unresolved problem, primarily due to the difficulty of discriminating between C–H bonds of comparable bond dissociation energies. Here, advances in polarity‐matched HAT catalysis and tunable radical mediators may offer a pathway forward, although competing pathways such as over‐oxidation and β‐scission will need to be carefully suppressed. Notably, photoredox catalysis provides a conceptual framework for addressing several of these challenges by decoupling radical generation from nitrate delivery, thereby enabling the controlled formation of reactive intermediates under mild conditions. In particular, the development of bifunctional reagents capable of orchestrating sequential radical formation and nitrate transfer within a single catalytic cycle represents a promising direction, especially when combined with electrochemical or flow‐based methodologies to enhance sustainability and scalability. Taken together, these advances suggest that the next phase of nitrooxylation chemistry will be defined by the transition from reagent‐driven reactivity toward catalyst‐controlled, modular platforms capable of delivering nitrate functionality with precision and predictability.

## Conflicts of Interest

The authors declare no conflicts of interest.
